# The Influence of the Topical Combined Application of Dexamethasone, Ascorbic Acid, and β‐Sodium Glycerophosphate on Implant Osseointegration: A Split‐Mouth Randomized Clinical Trial

**DOI:** 10.1155/ijod/5957866

**Published:** 2026-07-23

**Authors:** Manar Foura-Matar, Hazem Melad, Mohammed Taha

**Affiliations:** ^1^ Department of Oral and Maxillofacial Surgery, Faculty of Dentistry, Al-Azhar University-Gaza, Gaza, State of Palestine, alazhar.edu.ps; ^2^ Department of Pharmacology and Medical Sciences, Faculty of Pharmacy, Al-Azhar University-Gaza, Gaza, State of Palestine, alazhar.edu.ps

**Keywords:** β-sodium glycerophosphate, ascorbic acid, dexamethasone, immediate dental implants, implant stability, ISQ, osseointegration, osteogenic inducer

## Abstract

**Background:**

Immediate dental implant placement into extraction sockets offers reduced treatment time and favorable patient outcomes. Achieving early implant stability and predictable osseointegration is essential for long‐term success. This study aimed to evaluate whether a topical combination of dexamethasone, ascorbic acid, and β‐sodium glycerophosphate could enhance early implant stability in humans. The trial was designed as a randomized split‐mouth superiority study comparing test implants treated with the osteogenic inducer (OI) to control implants placed under identical surgical conditions.

**Methods:**

This split‐mouth randomized clinical trial included 18 systemically healthy adults requiring two adjacent or contralateral implants in the same jaw. All surgeries were performed at the Department of Oral and Maxillofacial Surgery, Al‐Azhar University‐Gaza. Each participant received one test implant (with a gelatin sponge saturated with the OI) and one control implant (with a plain gelatin sponge). Allocation of test and control sites was determined using centralized computer–generated randomization. The primary outcome was the implant stability, assessed using the Implant Stability Quotient (ISQ) at baseline and after 4, 8, and 16 weeks. Outcome assessment was performed by an examiner blinded to group allocation.

**Results:**

At 4 weeks, the test group demonstrated significantly higher ISQ values (70.19 ± 6.31) than the control group (65.35 ± 4.23; *p* = 0.036). No significant differences were observed at baseline, 8 weeks, or 16 weeks. However, implant stability increased significantly over time in both groups (*p* < 0.05), with the highest ISQ values observed at 16 weeks.

**Conclusion:**

The first‐in‐human clinical study investigating this topical osteogenic combination demonstrated a measurable short‐term benefit in enhancing early implant stability. Although the effect did not persist at later time points during the 16‐week follow‐up period, the findings suggest that this biological adjunct may support accelerated healing during the early phase of osseointegration.

**Trial Registration:**

ClinicalTrials.gov identifier: NCT07060859

## 1. Introduction

The use of dental implants and implant‐supported dentures has notably increased in recent decades due to an aging population and economic expansion [1]. A consistent goal in dental implantology has been to enhance osseointegration and reduce treatment time. Osseointegration, first described by Brånemark as a “direct structural and functional connection between ordered living bone and the surface of a load‐carrying implant” [2], is essential for clinical success [3]. However, primary stability remains critical to ensure a secure implant‐bone interface [4], and certain populations such as elderly, diabetic, and osteoporotic patients are more prone to implant failure [5–7].

Recent trends have emphasized the placement of immediate implants, which are inserted directly into extraction sockets [8–16]. These procedures often create peri‐implant gaps, commonly referred to as the “jumping distance,” which represents the horizontal space between the implant surface and the surrounding bone [17]. Such gaps may compromise early healing. Although bone grafting is common, slowly resorbing bone substitutes can delay healing [18, 19]. Therefore, interest has emerged in alternative, less invasive methods to promote faster osseointegration.

Studies have investigated the osteoinductive properties of dexamethasone, ascorbic acid, and β‐sodium glycerophosphate, particularly in promoting osteogenic differentiation of bone mesenchymal stem cells (BMSCs) [20, 21].

Dexamethasone, a synthetic glucocorticoid with well‐known anti‐inflammatory effects, also induces osteogenesis by activating alkaline phosphatase (ALP) and stimulating calcium deposition. Extensive evidence indicates that low‐dose dexamethasone enhances osteogenic differentiation of human bone‐marrow mesenchymal stem cells (hBMMCs), promoting calcium deposition and the formation of mineralized nodules. Furthermore, dexamethasone pretreatment has been shown to increase cellular responsiveness to major osteogenic regulators such as BMP‐2 and BMP‐6, which act synergistically to promote bone formation [22, 23]. Its osteogenic effect is dose‐dependent, with optimal activity consistently reported at the physiological concentration range of 10^−8^–10^−10^ mol/L; however, higher doses may suppress MSC proliferation and differentiation [24].

Ascorbic acid (vitamin C) contributes to collagen synthesis and extracellular matrix formation, enhancing osteoblast proliferation, ALP activity, and mineralization, especially when combined with growth factors [21, 25, 26]. β‐Sodium glycerophosphate provides phosphate ions essential for the mineralization of calcium salts, accelerating bone formation [27].

The mechanism of action is likely associated with the interplay among dexamethasone, ascorbic acid, and β‐sodium glycerophosphate, which synergistically facilitate osteoblast proliferation and differentiation, enhance alveolar wound healing, and reduce alveolar bone resorption. It is widely accepted that successful bone formation requires three essential components: an appropriate biological environment, the presence of responsive target cells, and osteoinductive signals [28]. A practical advantage of the alveolar socket is the abundance and accessibility of resident bone‐ forming cells compared with other potential cell sources, without exposing the patient to additional surgical risks [29]. While their efficacy is well‐documented in vitro and in animal models [22, 23], clinical evidence in humans remains limited.

This study is therefore significant, as existing bone regeneration strategies are often expensive, technically demanding, and carry risks such as contamination [30, 31]. No consensus exists regarding the optimal biomaterial or protocol for enhancing healing. A simple, cost‐effective, and biocompatible approach, such as the local application of an osteogenic inducer (OI) via a gelatin sponge, may offer improved outcomes, particularly in patients with compromised bone healing.

The objective of this split‐mouth randomized clinical trial was to evaluate the effect of topically applied dexamethasone, ascorbic acid, and β‐sodium glycerophosphate on implant osseointegration and stability, using a gelatin sponge as a sustained‐release carrier during the healing period. The null hypothesis was that there would be no significant difference in implant stability (implant stability quotient (ISQ) values) between implants treated with the OI and untreated control implants.

## 2. Materials and Methods

### 2.1. Study Design

A single‐blinded split‐mouth randomized clinical trial was conducted at the Faculty of Dentistry clinics, Al‐Azhar University– Gaza, between January and September 2023. This study followed a randomized split‐mouth design, in which each patient received two implants within the same jaw (either maxilla or mandible). One implant site was randomly allocated to the test condition with topical application of the OI, while the adjacent or contralateral site in the same arch was assigned as the control condition without the OI (plain sponge). The study was conducted and reported in accordance with the CONSORT 2025 statement. The CONSORT 2025 checklist and participant flow diagram are provided in Supporting Information.

### 2.2. Study Setting and Duration

The trial was conducted at the Department of Oral and Maxillofacial Surgery clinics, Faculty of Dentistry, Al‐Azhar University–Gaza. Eligible participants were evaluated and recruited through the faculty’s outpatient clinics. The total duration of follow‐up per patient was 16 weeks, with implant stability measurements at 0, 4, 8, and 16 weeks. All surgical procedures were performed by a single experienced oral surgeon trained in implant placement to ensure consistency and standardization of the intervention across participants.

### 2.3. Sample Size and Randomization

The sample size was calculated using a paired *t*‐test, with a significance level of 0.05, a power of 0.8, and an effect size of 0.5. Based on these parameters (*σ* = 0.69, *d* = 0.5, *Zα*/2 = 1.96, and *Zβ* = 0.84), the minimum required sample size was ~15 participants. A total of 18 patients were ultimately enrolled. Random assignment of test and control implant sites was performed within each patient. Randomization was performed using a computer‐generated sequence, with allocation concealed in sealed opaque envelopes and revealed at the time of surgery.

### 2.4. Inclusion and Exclusion Criteria

#### 2.4.1. Inclusion Criteria

Patients aged ≥18 years, good general health, presence of two unrestorable teeth requiring extraction and immediate implant insertion within the same jaw (either maxilla or mandible), positioned either adjacent or contralateral to each other, willingness to provide informed consent, and availability for all study procedures including CBCT.

#### 2.4.2. Exclusion Criteria

Systemic disease affecting bone turnover, contraindications for surgery, psychiatric illness, or chronic medication use affecting bone, and current cigarette smoking.

### 2.5. Ethical Approval

This study was conducted in accordance with the Declaration of Helsinki and was approved by the Helsinki Committee for Ethical Approval at the Palestinian Health Research Council (PHRC), Gaza Strip, Palestine (Approval Number PHRC/HC/1209/22, December 5, 2022). Additional approval was obtained from the Al‐Azhar University Scientific Committee. The OI specimens were stored at −20 to −55°C under the approval of the Palestinian Medical Relief Society (PMRS). Written informed consent was obtained from all participants prior to enrollment.

### 2.6. Study Instruments and Drug Preparation

**Figure 1:**
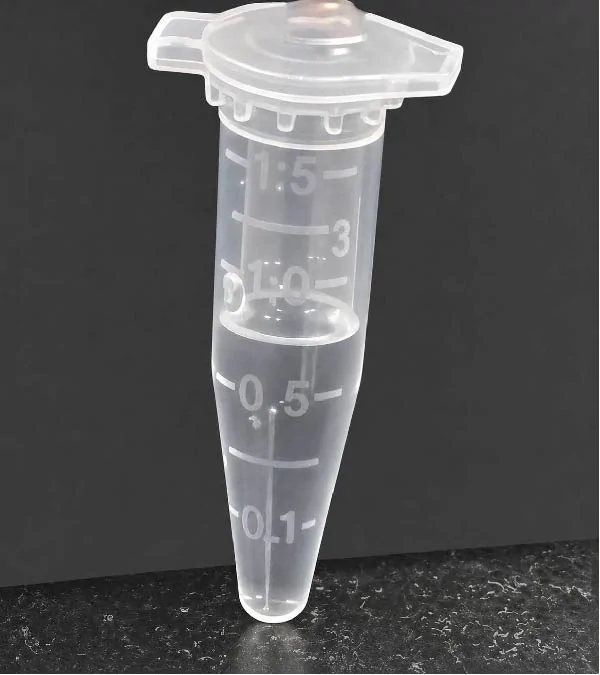
The aliquoted osteogenic inducer, divided according to the maximum absorption capacity of the gelatin sponge per case.

The OI preparation followed the protocol of Chen et al. [27]. The OI solution was prepared by combining dexamethasone (10^−8^ mol/L; Sigma‐Aldrich–Merk, Ramallah, #D4902), β‐sodium glycerophosphate (10 mmol/L; Sigma‐Aldrich–Merk, Ramallah, #G9422), and L‐ascorbic acid (50 mg/L; Megapharm, Gaza). For accuracy, each component was initially prepared as a concentrated stock solution, followed by serial dilutions to achieve the desired final concentrations. The individual solutions were then combined to reach a final volume of 510 mL. The mixture was sterilized using a 0.22 µm filter, aliquoted into sterile containers (Figure 1), and stored at −20 to −55°C until use. For application, a gelatin sponge (5 × 15 × 10 mm; Jinling Pharmaceutical, China) was soaked in the OI solution, absorbing ~75 mg of the osteogenic solution, with absorption verified by pre‐ and postsoaking weights. Control sponges were prepared identically but without active ingredients.

### 2.7. Surgical Procedures

**Figure 2:**
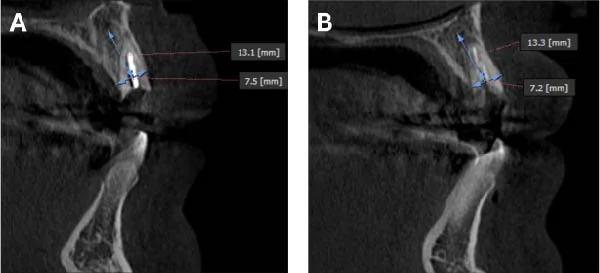
CBCT analysis of the alveolar ridge dimensions at the implant sites. (A) Upper right central incisor (#11) and (B) upper right lateral incisor (#12).

Teeth were atraumatically extracted under local anesthesia using 2% lidocaine with 1:100,000 epinephrine (Septodont Ltd., UK). Pyramidal flaps were raised, and the extraction sockets were curetted and irrigated with sterile saline. Osteotomies were prepared following B&B Dental implant protocols, with implant dimensions selected individually according to each patient’s anatomical conditions. The implant size varied between patients; however, in accordance with the split‐mouth design, each patient received implants of identical dimensions on both sides (Figure 2A, B).

**Figure 3:**
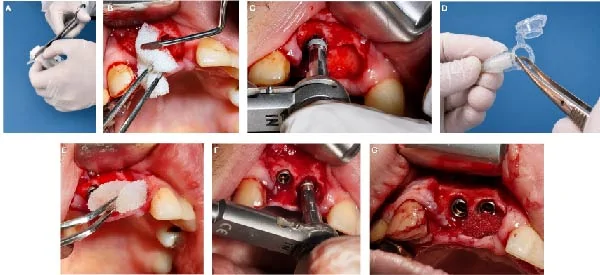
(A) A gelatin sponge was measured, cut, and designed according to the appropriate measurements for placement in the control osteotomy implant site. (B) Adaptation of plain gelatin sponge in the control osteotomy implant site. (C) Insertion of (4 Ø × 12 mm) implant in the control gelatin sponge osteotomy site. (D) A gelatin sponge was measured, cut, designed, and immersed in the OI solution for placement in the test osteotomy implant site. (E) Adaptation of OI gelatin sponge in the test osteotomy implant site. (F) Insertion of (4 Ø × 12 mm) implant in the test OI osteotomy site. (G) Clinical view showing both implants in their correct positions.

In the test sites, a gelatin sponge previously soaked with the OI solution was inserted into the socket before implant placement. In the control sites, a plain gelatin sponge without the OI was inserted in the same manner. The implants were then placed accordingly, and healing abutments were attached (Figure 3A–G).

### 2.8. Implant Placement and Stability Measurement

**Figure 4:**
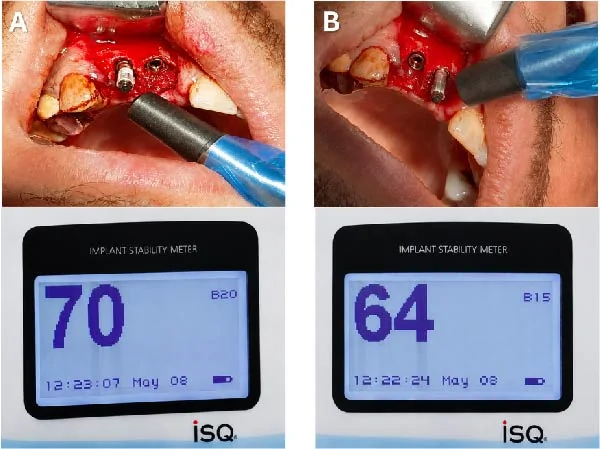
Stability measurements recording by using Osstell device: (A) for the control implant (GS) and (B) for the test implant (OI).

Dental implants (Dura‐Vit 3P Line; B&B Dental, Bologna, Italy) with a double‐etched microrough surface were used. Implant stability measurements were obtained using a resonance frequency analysis device (Osstell ISQ, Model 100500; Osstell AB, Gothenburg, Sweden) with the corresponding SmartPeg and ISQ probe (Ref. 100501), according to the manufacturer’s instructions. ISQ values (range: 1–100) were recorded in both the buccolingual and mesiodistal directions, and the mean value was calculated for analysis (Figure 4A, B). All measurements were performed by an examiner blinded to test and control allocation.

### 2.9. Postoperative Care and Follow‐Up

Patients received amoxicillin/clavulanic acid (875/125 mg), ibuprofen (600 mg), and chlorhexidine mouthwash postoperatively. Sutures were removed after 1 week. Implants were loaded prosthetically after 16 weeks. Each implant was individually followed throughout the study, with minor visit timing deviations (±3 days) permitted. At each follow‐up visit, clinical and radiographic assessments were performed. The implants were prosthetically loaded after 16 weeks. Participants were monitored for postoperative complications, including infection, excessive pain, swelling, or implant failure. No serious adverse events were observed during the study.

### 2.10. Outcomes

The primary outcome of the present study was the longitudinal change in implant stability, quantified by the ISQ at baseline and subsequently at 4, 8, and 16 weeks postoperatively. The secondary outcomes comprised the implant survival rate and the assessment of peri‐implant clinical health parameters, including the absence of pain, infection, inflammation, and any detectable mobility of the implant.

### 2.11. Variables and Statistical Analysis

Data were analyzed to test the null hypothesis that no significant difference in implant stability (ISQ values) exists between the test (OI) and control implants over time. The primary outcome was implant stability, measured by the ISQ at baseline, 4, 8, and 16 weeks. The independent variables included the treatment group (test or control) and time point.

Statistical analyses were performed using the R software (version 4.4.0). Data normality was assessed using the Shapiro–Wilk test, and the homogeneity of variances was evaluated with Levene’s test. A linear mixed‐effects model was applied to account for the repeated‐measures split‐mouth design. The false discovery rate (FDR) correction was used to adjust for multiple comparisons. Statistical significance was set at *p* < 0.05. No missing data were observed, and all values were included in the analysis.

## 3. Results

### 3.1. Demographic Data

Eighteen patients participated in the study (mean age: 37.39 ± 6.85 years), including eight males (44.44%) and 10 females (55.56%) as presented in Table 1.

**Table 1:** Summary statistics for demographic data.

Parameter		Value
Gender [*n* (%)]	Male	8 (44.44%)
	Female	10 (55.56%)
Age (years) (mean ± SD)		37.39 ± 6.85

### 3.2. Implant Distribution and Characteristics

The distribution and characteristics of the implants are presented in Table 2. In this split‐mouth study, each patient received one test and one control implant placed within the same jaw. Implant positions included central incisors, lateral incisors, canines, and premolars, with comparable distribution between test and control implants. The implant diameter and length were also matched within each patient. The distribution between the maxilla and mandible was balanced across groups (10 maxillary and eight mandibular implants per group).

**Table 2:** Implant distribution and characteristics in test and control groups.

Patient ID	Test implant (*n* = 18)				Control implant (*n* = 18)			
	Jaw	Site	Diameter (mm)	Length (mm)	Jaw	Site	Diameter (mm)	Length (mm)
1	Mx	11	4.0	12	Mx	21	4.0	12
2	Mx	11	4.0	12	Mx	21	4.0	12
3	Mx	21	4.0	12	Mx	11	4.0	12
4	Mx	11	4.0	12	Mx	21	4.0	12
5	Mx	11	3.5	12	Mx	12	3.5	12
6	Mx	12	3.5	12	Mx	22	3.5	12
7	Md	42	3.5	12	Md	32	3.5	12
8	Mx	12	4.0	12	Mx	11	4.0	12
9	Mx	12	4.0	12	Mx	21	4.0	12
10	Mx	23	4.0	12	Mx	13	4.0	12
11	Md	43	4.0	12	Md	33	4.0	12
12	Md	43	4.0	12	Md	33	4.0	12
13	Md	43	4.0	12	Md	44	4.0	12
14	Md	45	4.0	12	Md	44	4.0	12
15	Mx	14	4.0	12	Mx	13	4.0	12
16	Md	45	4.0	10	Md	44	4.0	10
17	Md	35	4.0	10	Md	34	4.0	10
18	Md	45	4.5	12	Md	44	4.5	12

*Note:* Tooth numbering follows the FDI two‐digit system.

Abbreviations: Md, mandible; Mx, maxilla.

### 3.3. Implant Survival

All implants were followed according to the planned schedule for 16 weeks. One paired set of implants (test and control) failed before the 16‐week follow‐up evaluation. ISQ measurements for both implants were recorded up to the point of failure and included in the statistical analysis. No additional complications or losses to follow‐up were observed.

### 3.4. Descriptive Statistics

Implant stability (ISQ) was recorded at four time points. At baseline, the test group (OI) had a mean ISQ of 66.08 ± 8.69, while the control group (GS) had a higher mean of 68.20 ± 5.47. At 4 weeks, the test group demonstrated higher stability (70.19 ± 6.31) compared to the control group (65.35 ± 4.23). At 8 weeks, the mean ISQ values were 70.93 ± 5.05 for the test group and 68.80 ± 6.01 for the control group. At 16 weeks, both groups showed similar ISQ values: 73.77 ± 4.94 (test) and 74.08 ± 4.82 (control). Descriptive statistics for implant stability (ISQ) values are presented in Table 3.

**Table 3:** Descriptive statistics for the implant stability (ISQ).

Time	Group	Mean	95% confidence interval		SD	Min.	Max.
			Lower	Upper			
Baseline	Test	66.08	61.35	70.80	8.69	49.00	81.00
	Control	68.20	65.23	71.18	5.47	58.00	77.00
4 weeks	Test	70.19	66.76	73.62	6.31	60.00	83.00
	Control	65.35	63.05	67.65	4.23	58.00	73.00
8 weeks	Test	70.93	68.19	73.68	5.05	59.00	79.00
	Control	68.80	65.54	72.07	6.01	60.00	80.00
16 weeks	Test	73.77	71.09	76.45	4.94	66.00	81.00
	Control	74.08	71.46	76.70	4.82	68.00	82.00

### 3.5. Effect of Variables and Interaction

Only measurement time showed a statistically significant effect on ISQ values (*p* = 0.006). The OI itself (*p* = 0.152) and its interaction with time (*p* = 0.070) were not statistically significant. The effects of different variables and their interactions on implant stability (ISQ) are presented in Table 4.

**Table 4:** Effect of different variables and their interactions on implant stability (ISQ).

Source	Sum of squares (II)	df	Mean square	*f*‐Value	*p*‐Value
OI	33.47	1	33.47	2.35	0.152^ns^
Time	731.82	3	243.94	**4.88**	**0.** **006** ^∗^
Group ^∗^ time	178.25	3	59.42	2.57	0.070^ns^

*Note:* The bold values were intended to highlight statistically significant results.

Abbreviations: df, degree of freedom; ns, nonsignificant.

^∗^Significant (*p* < 0.05).

### 3.6. Effect of OI

**Figure 5:**
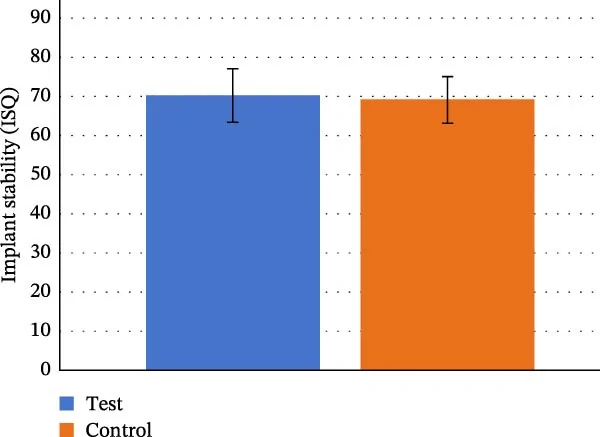
Bar chart showing mean implant stability (ISQ) values with standard deviation error bars for the test (OI) and control (GS) groups.

Across all time points, the test group exhibited slightly higher mean stability (70.24 ± 6.83) compared to the control group (69.11 ± 5.94), although the difference was not statistically significant (*p* = 0.152) as presented in Table 5 and Figure 5.

**Table 5:** Intergroup comparisons and summary statistics of implant stability (ISQ).

Implant stability (ISQ) (mean ± SD)		*p*‐Value
Test (OI)	Control (GS)	
70.24 ± 6.83	69.11 ± 5.94	0.152^ns^

Abbreviation: ns, nonsignificant.

### 3.7. Effect of Time

**Figure 6:**
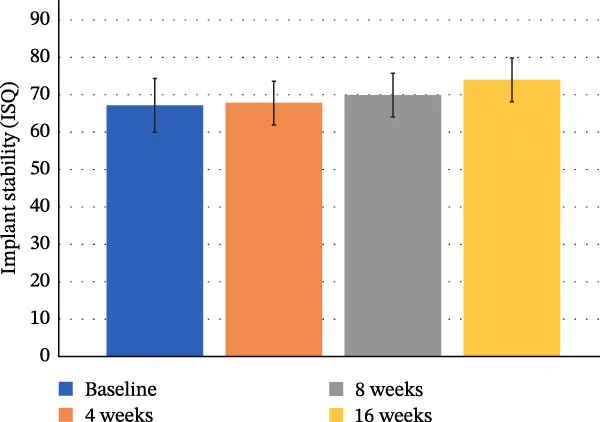
Bar chart showing the intragroup comparison of mean implant stability (ISQ) values with standard deviation error bars at baseline, 4 weeks, 8 weeks, and 16 weeks in the test (OI) group.

A significant increase in stability was observed over time (*p* = 0.006). The ISQ values increased from 67.14 ± 7.20 at baseline to 73.92 ± 4.78 at 16 weeks (Table 6). Pairwise comparisons showed significant differences between the 16‐week values and those at baseline and 4 weeks (Figure 6).

**Table 6:** Intragroup comparison and summary statistics of implant stability (ISQ).

Implant stability (ISQ) (mean ± SD)				*p*‐Value
Baseline	4 weeks	8 weeks	16 weeks	
67.14 ± 7.20^B^	67.77 ± 5.81^B^	69.87 ± 5.55^AB^	73.92 ± 4.78^A^	**0.006** ^∗^

*Note:* Values with different superscripts within the same horizontal row are significantly different. The bold values were intended to highlight statistically significant results.

^∗^Significant (*p* < 0.05).

### 3.8. Interaction Between Group and Time

**Figure 7:**
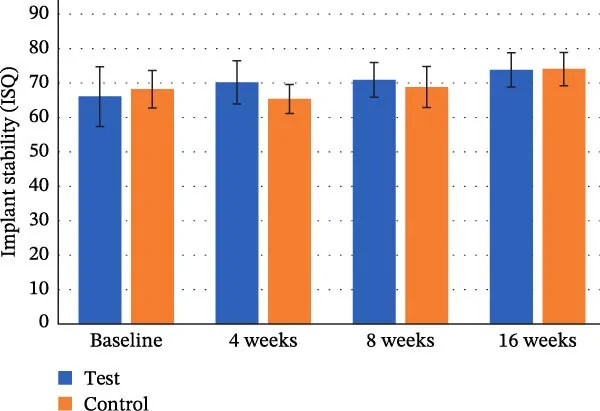
Bar chart showing mean implant stability (ISQ) values with standard deviation error bars in the test (OI) and control (GS) groups at baseline, 4 weeks, 8 weeks, and 16 weeks.

Table 7 shows that at 4 weeks, the test group showed significantly higher stability than the control group (*p* = 0.036). No other time point comparisons between groups were statistically significant. Within‐group comparisons revealed significant improvements over time in both groups: the test group (*p* = 0.011) and the control group (*p* = 0.003), particularly between baseline and 16 weeks (Figure 7).

**Table 7:** Inter‐ and intragroup comparisons and summary statistics of implant stability (ISQ).

OI	Implant stability (ISQ) (mean ± SD)		*p*‐Value
Time	Test	Control	
Baseline	66.08 ± 8.69^B^	68.20 ± 5.47^AB^	0.351^ns^
4 weeks	70.19 ± 6.31^AB^	65.35 ± 4.23^B^	**0.036** ^∗^
8 weeks	70.93 ± 5.05^AB^	68.80 ± 6.01^AB^	0.350^ns^
16 weeks	73.77 ± 4.94^A^	74.08 ± 4.82^A^	0.892^ns^
*p*‐Value	**0.011** ^∗^	**0.003** ^∗^	

*Note:* Values with different superscripts within the same vertical column are significantly different. The bold values were intended to highlight statistically significant results.

Abbreviation: ns, nonsignificant.

^∗^Significant (*p* < 0.05).

**Figure 8:**
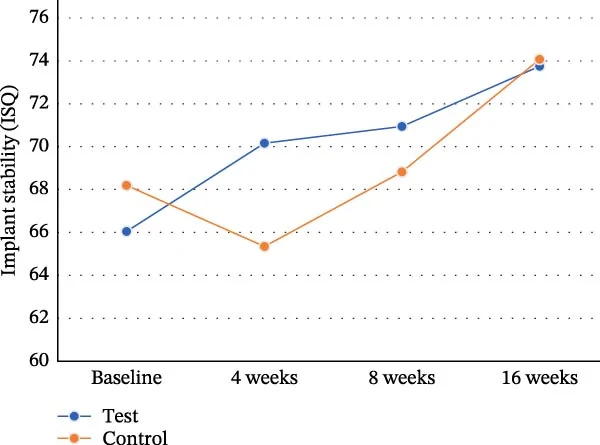
Line chart showing average implant stability (ISQ).

These findings indicate a general improvement in implant stability over time, with transient superiority of the OI group at 4 weeks (Figure 8).

## 4. Discussion

This study evaluated the effect of a topical combination of dexamethasone, ascorbic acid, and β‐sodium glycerophosphate on implant osseointegration. A total of 36 implants were placed in 18 patients, with each patient receiving both a test (OI‐treated) and control (untreated) implant. In this study, both test and control implants for each patient were placed within the same jaw (either maxilla or mandible), positioned either adjacent or contralateral to each other. This design ensured that ISQ comparisons reflected the effect of the OI rather than anatomical differences in bone density between the maxilla and mandible, which are known to influence implant stability values [32]. Implant stability was measured over a 16‐week period.

The follow‐up intervals at 0, 4, 8, and 16 weeks were selected to capture the progression of the early and preloading phases of the osseointegration process. At the outset, it was uncertain to what extent the effect of the OI would persist. Therefore, the follow‐up period was extended slightly beyond the initial healing phase to monitor whether any changes continued toward the maturation stage prior to loading.

The results showed a significant increase in implant stability over time in both groups, aligning with the natural progression of osseointegration. Notably, the test group exhibited a significantly higher ISQ at 4 weeks, indicating enhanced early bone healing, though no significant differences were observed at 8 or 16 weeks.

Our findings are consistent with Chen et al. [27], who demonstrated enhanced bone formation using the same osteogenic combination in extraction sockets. Similarly, Chen et al. [1] found that local application of OIs using a sustained‐release system improved bone remodeling and implant integration in animal models. Das et al. [33] supported these outcomes by showing improved osseointegration through nanofibrous coatings incorporating dexamethasone, promoting mesenchymal stem cell differentiation.

The early ISQ gain at 4 weeks in our study also aligns with reports from Jaiswal et al. [34] and Akahane et al. [35], emphasizing the synergy of dexamethasone, ascorbic acid, and β‐glycerophosphate in stimulating osteogenesis [20, 24, 29, 34–39]. The observed enhancement in implant osseointegration can be attributed to the synergistic biological effects of the three compounds. Dexamethasone promotes osteoblast differentiation by upregulating key transcription factors such as Runx2 and Osterix, and it enhances ALP activity and mineralized matrix deposition [20, 34, 40, 41]. Ascorbic acid acts as a cofactor for collagen synthesis and stimulates osteogenic genes related to osteoblast differentiation and maturation, such as Runx2, Osterix, and osteocalcin, contributing to extracellular matrix mineralization and bone healing [42–46]. β‐Sodium glycerophosphate provides essential phosphate ions for hydroxyapatite formation and enhances ALP expression, further supporting matrix mineralization [47–50]. The combined topical use of these agents has been shown to synergistically improve implant integration [1].

The extremely low dose of dexamethasone used in this formulation (10^−8^ mol/L), combined with its localized delivery within a gelatin sponge confined to the implant site, suggests minimal systemic absorption, consistent with previous reports of negligible systemic exposure following low‐dose, locally applied corticosteroids in dental tissues [1, 24, 27, 51–57]. Pharmacokinetic data from microdose dexamethasone delivery systems further confirm that localized administration results in extremely low plasma concentrations, well below thresholds associated with systemic corticosteroid activity [58, 59].

The findings of previous preclinical studies support the biological plausibility of the transient improvement observed in our trial [24, 27]. In the study by Liu et al. [24], OI‐impregnated gelatin sponges implanted into rabbit extraction sockets significantly enhanced socket healing, a result consistent with earlier evidence reported by Chen et al. [27]. Given that the biological sequence of implant healing closely parallels that of extraction socket healing, these studies suggested that OI may similarly enhance early osseointegration around implants. Additional data from the same research group demonstrated that OI reduced vertical alveolar bone resorption, although changes in ridge width were not significant—an effect attributed to the relatively short half‐life of the OI when delivered via gelatin sponges [60].

In line with these findings, our results also revealed an early but temporary improvement in implant stability, which subsequently diminished over time. This transient effect is likely related to the biodegradation of the gelatin sponge carrier [61] and the overriding influence of natural bone remodeling processes after ~8 weeks [62]. The significant difference in ISQ at 4 weeks may reflect an acceleration of the dip stage recovery, which is critical during early implant healing, by enhancing new bone formation. By weeks 8 and 16, implant stability became comparable between the test and control groups. This convergence may be attributed to a biological plateau, where the initial stimulatory effects of the OI reach a temporal threshold [62]. Once early bone formation is initiated, subsequent improvements in implant stability are predominantly governed by natural bone remodeling and maturation processes at the bone‐implant interface, rather than continued activity of the applied agents.

In addition, mechanical factors such as implant macro‐ and microdesign, surface topography, and occlusal loading conditions become increasingly influential in maintaining stability during the late stages of osseointegration. These parameters may surpass the early biochemical influence of OIs, as reported in previous literature [63].

The implant site has been reported to influence primary stability and ISQ values, largely due to anatomical variations in bone density between different tooth positions [64, 65]. For example, posterior sites often show higher ISQ values compared with anterior regions, reflecting differences in cortical bone support. However, when both test and control implants are placed within the same jaw of the same patient, as in the present split‐mouth design, the influence of site variability is minimized because intrapatient comparisons are made under similar anatomical conditions. Furthermore, implant diameter and length were matched within each patient. As detailed in Table 2, the distribution of implant sites between test and control implants was comparable, reducing the likelihood that implant location confounded the intrapatient comparisons. Therefore, the observed differences in early stability are more likely attributable to the OI rather than implant location.

More recently, Kerem et al. [66] demonstrated the bioactivity enhancement of titanium‐based implants through chitosan microsphere coatings loaded with dexamethasone. The modified surfaces improved roughness and cell adhesion, while controlled drug release supported early osteogenic differentiation and mineral deposition. These results are consistent with the broader principle of using bioactive coatings or local therapeutic applications to accelerate and improve osseointegration. Clinically, such an inducer could be beneficial in cases requiring early loading or in patients with impaired bone healing.

Although direct comparisons remain limited due to the unique formulation used in the present study, the overall strategy of locally enhancing the healing environment aligns well with existing research on biomaterial functionalization and localized drug delivery approaches.

CBCT imaging can provide valuable information regarding peri‐implant bone morphology and buccolingual width; however, its routine use was not incorporated into the present study. This decision was based on current clinical guidelines, which recommend limiting CBCT to situations involving specific complications or diagnostic uncertainty in order to avoid unnecessary radiation exposure when no clear clinical indication exists [67–71]. In addition, CBCT has limited sensitivity for detecting subtle early‐stage bone changes, and its diagnostic performance may be further reduced by scattering and beam‐hardening artifacts that can obscure shallow defects and early peri‐implant radiolucency. Recent reviews have also concluded that CBCT cannot yet be considered a standard postoperative tool for evaluating peri‐implant bone, despite its advantages in buccolingual visualization and three‐dimensional assessment [72]. In this context, ISQ was considered an appropriate primary tool, as it offers a validated, noninvasive, and reproducible method for evaluating implant stability without exposing patients to radiation.

Nonetheless, the study has certain limitations. The relatively small sample size may affect the generalizability of the findings, and relying solely on ISQ means that direct volumetric information on peri‐implant bone morphology was not obtained. Future studies would benefit from larger cohorts, exploration of alternative sustained‐release formulations of OIs, incorporation of complementary diagnostic methods, and evaluation of additional parameters such as insertion torque to further elucidate factors influencing primary implant stability.

## 5. Conclusion

This study demonstrated that a topical combination of dexamethasone, ascorbic acid, and β‐sodium glycerophosphate was associated with higher implant stability values during the early healing phase, with a significant difference observed at 4 weeks postoperatively. By 16 weeks, stability values in both groups were comparable, suggesting that the observed effect was limited to the initial phase of healing. These findings suggest that this topical approach may serve as a potential adjunct for supporting early osseointegration; however, its long‐term clinical relevance requires confirmation through studies with extended follow‐up periods.

### 5.1. Recommendations

• Larger‐scale randomized multicenter trials should be conducted to further validate the current findings and enhance the generalizability of the results.
• Long‐term follow‐up studies are required to determine whether the early stability improvements observed in this trial translate into sustained implant success over time.
• Advanced delivery systems for the sustained and targeted release of osteogenic agents should be explored to improve therapeutic efficacy.
• The underlying biological mechanisms should be investigated through in‐depth cellular and molecular studies.
• Standardized clinical protocols for the application of osteoinductive compounds should be developed to ensure consistency and safety.
• The potential use of these agents in medically compromised patients and across broader regenerative fields should be evaluated.

## Author Contributions

**Manar Foura-Matar**: conceptualization, methodology, investigation, clinical implementation of the study, implant placement surgeries, patient recruitment and management, resource provision, formal analysis, writing – original draft preparation, manuscript revision, project administration, overall coordination of the study. **Hazem Melad**: methodology, supervision, oversight of the study implementation, supervision of the clinical procedures and surgical interventions, validation of the results, review of tables and figures, literature review, writing – review and editing, overall supervision of the project from inception to completion. **Mohammed Taha**: preparation of the osteogenic inducer solution, supervision of its aliquoting and refrigerated storage procedures, literature review and scientific editing related to the study topic, scientific support.

## Funding

This research received no external funding.

## Conflicts of Interest

The authors declare no conflicts of interest.

## Supporting Information

Additional supporting information can be found online in the Supporting Information section.

## Supporting information


**Supporting Information** CONSORT 2025 checklist and participant flow diagram. This Supporting Information is available online with the published article.

## Data Availability

The data that support the findings of this study are available from the corresponding author upon reasonable request.
