# Supplementary material for: The Influence of the Topical Combined Application of Dexamethasone, Ascorbic Acid, and β‐Sodium Glycerophosphate on Implant Osseointegration: A Split‐Mouth Randomized Clinical Trial
Source: Int J Dent. 2026 Jul 23;2026:5957866. doi: 10.1155/ijod/5957866 (PMC13392916; doi:10.1155/ijod/5957866)
Supplement: Supplementary file 1 — Supporting Information 1 Material 1 (Sample size calculations). Material 2 (Preparation of osteogenic inducer solution). Material 3 (Study hypotheses). Material 4 (Follow‐up schedule). Figures S9–S10 (Ethical and administrative approvals). Forms 1–2 (Informed consent forms). File 1 (Safety data sheet: Dexamethasone). File 2 (Certificate of analysis: Dexamethasone). File 3 (Safety data sheet: β‐Glycerophosphate disodium salt hydrate). File 4 (Certificate of analysis: β‐Glycerophosphate disodium salt hydrate). File 5 (USP monograph: Ascorbic acid). This Supporting Information is available online with the corresponding author upon reasonable request. [file IJOD-2026-5957866-s001.docx]

**Supplementary Materials**

[***Supplementary Material 1:*** Sample Size Calculations 2](#_Toc214740566)

***[Supplementary Material 2:](#_Toc214740567)*** [Preparation of Osteogenic Inducer Solution 3](#_Toc214740567)

***[Supplementary Material 3:](#_Toc214740568)*** [Study Hypotheses 12](#_Toc214740568)

[***Supplementary Material 4:*** Detailed follow-up schedule for each implant 13](#_Toc214740569)

[***Supplementary Figure S9*:** Ethical Approval of Helsinki Committee of the Palestinian Health Research Council (PHRC) 14](#_Toc214740570)

[***Supplementary Figure S10:*** Approval of The Palestinian Medical Relief Society (PMRS) 15](#_Toc214740571)

[***Supplementary Form 1 –*** Informed Consent Form (Arabic) 16](#_Toc214740572)

[***Supplementary Form 2 –*** Informed Consent Form (English) 17](#_Toc214740573)

[***Supplementary File 1 –*** Safety Data Sheet: Dexamethasone (D4902***)*** 18](#_Toc214740574)

[***Supplementary File 2 –*** Certificate of Analysis: Dexamethasone (Batch BCCJ0003) 27](#_Toc214740575)

[***Supplementary File 3 –*** Safety Data Sheet: β-Glycerophosphate disodium salt hydrate (G9422) 28](#_Toc214740576)

[***Supplementary File 4 –*** Certificate of Analysis: β-Glycerophosphate disodium salt hydrate (Batch SLCP2510***)*** 35](#_Toc214740577)

[***Supplementary File 5 –*** USP Monograph: Ascorbic Acid 37](#_Toc214740578)

***Supplementary Material 1: Sample Size Calculations***

Given an experimental design, where each patient has two implants (one test and one control), a paired t-test used to calculate the required sample size. In this scenario, the analysis considers the within-subject differences between the test and control implants.


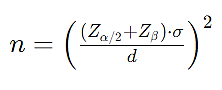


*where:*

- Standard deviation (σ) ≈ 0.69
- Significance level (α) = 0.05

For α=0.05, the critical value 𝑧𝛼/2 can be found from the standard normal distribution. Since it's a two-tailed test, 𝑧 𝛼/2 ≈1.96

- Type II error (β) = 0.2 (for a power of 0.8)

For 𝛽 = 0.2, the critical value 𝑧𝛽 for power (1 - *β* = 0.8) can be found from the standard normal distribution. 𝑧𝛽≈0.84

- Effect size (d) = 0.5

*Plugging the values into the formula:*


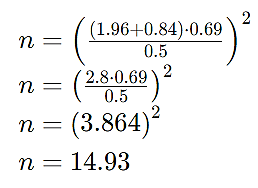


So, approximately ***15 participants*** total (since each participant serves as their own control).

***Supplementary Material 2: Preparation of Osteogenic Inducer Solution***

To prepare the osteogenic inducer solution, the following steps were undertaken:

1. **Preparation of 10⁻⁸ mol/L dexamethasone solution**

*Dexamethasone (formula: C_22_H_29_FO_5_, molecular weight: 392.46 g/mol)*

**Step 1:** The required amount of dexamethasone for a 10⁻⁸ mol/L solution was calculated:


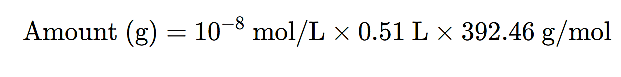


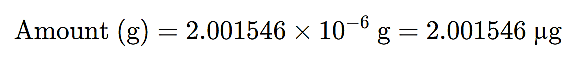


The required amount of dexamethasone for the solution is 2.001546 µg. However, weighing such a small amount accurately is challenging. Therefore, a stock solution with a higher concentration was typically prepared first and then diluted to the desired concentration.

**Step 2:** Pre-weighted 25 mg of dexamethasone were dissolved in 510 mL of methanol to prepare a stock solution with a concentration of:


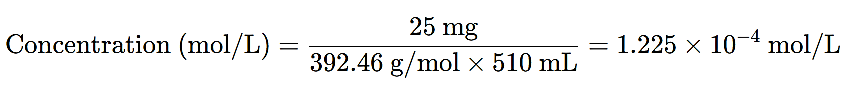


**Step 3:** To prepare the final 10⁻⁸ mol/L solution, the stock solution accordingly was diluted accordingly:


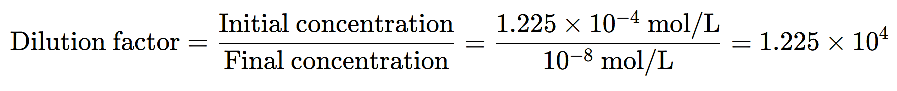


**Step 4:** The volume of the stock solution needed to achieve the desired concentration in 510 mL of the total combination volume:


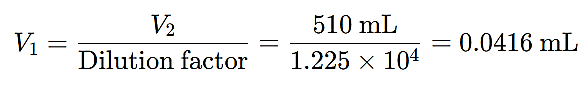


- **Practical Steps:**

1. 0.0416 mL (41.6 µL) of the 1.225 × 10⁻⁴ mol/L stock solution was measured.
2. The 41.6 µL of the stock solution with methanol was diluted to a final volume of 510 mL to achieve the desired 10⁻⁸ mol/L dexamethasone solution.


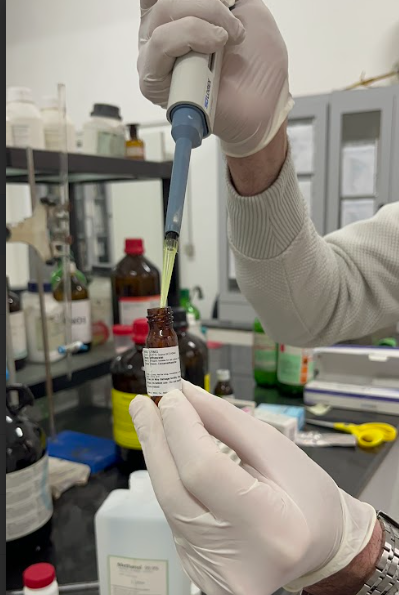


**B**


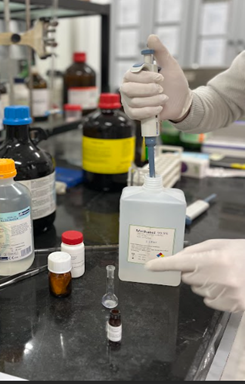


**A**


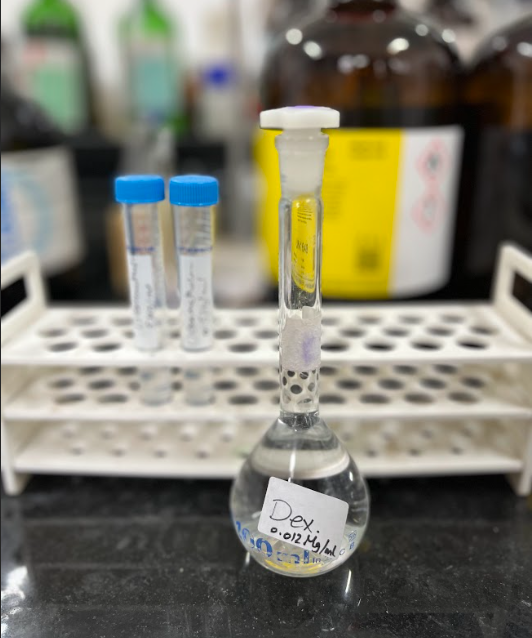


**C**

**Supplementary Figure S1. A.** Measurement of methanol solvent, **B.** Dissolving of dexamethasone powder, **C.** The prepared volume of dexamethasone solution.

1. **Preparation of 10 mmol/L β-Sodium Glycerophosphate Solution**

*β-sodium glycerophosphate (formula: C_3_H_7_Na_2_O_6_P.xH_2_O, molecular weight: 216.04 g/mol).*

**Step 1:** The required amount of β-sodium glycerophosphate for a 10 mmol/L solution was calculated:


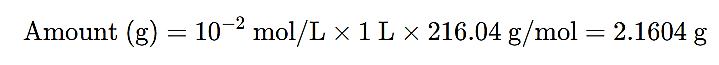


**Step 2:** 1629.2 **mg of β-Sodium Glycerophosphate powder was accurately weighed and dissolved in 50 mL of distilled water until fully dissolved.**

**Step 3: This concentrated solution needed to be further diluted to achieve the desired concentration:**

To prepare the 10 mmol/L solution from the 1.6292 g dissolved in 50 mL:

- The concentration of the initial solution was calculated:


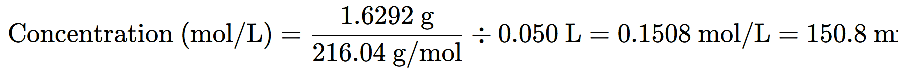


- Dilute the initial solution to achieve a 10 mmol/L concentration using the dilution factor:


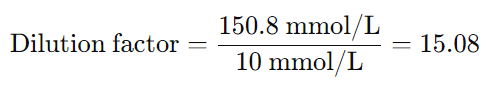


- The final volume (510 mL) was used:


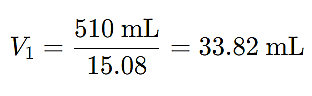


- **Practical Steps:**

1. 1629.2 mg (1.6292 g) of β-Sodium Glycerophosphate powder accurately weighted, **Figure (2, A).**
2. The 1.6292 g were dissolved in 50 mL of distilled water to make an initial concentrated solution (150.8 mmol/L), **Figure (2, B and C).**
3. 33.82 mL of this concentrated solution were taken.
4. The 33.82 mL of the initial solution were diluted to a final volume of 510 mL with distilled water to achieve a 10 mmol/L β-Sodium Glycerophosphate solution.


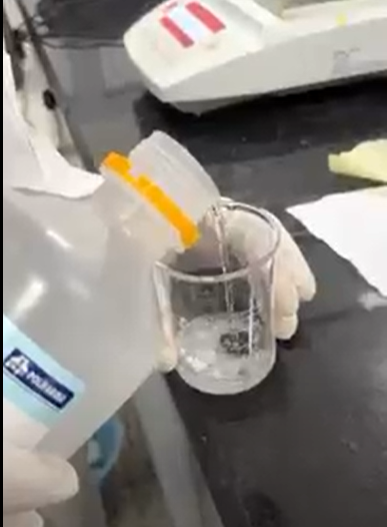


**B**


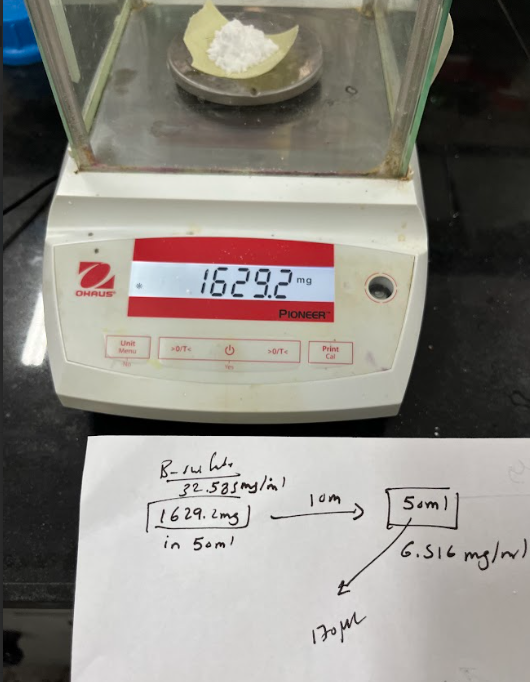


**A**


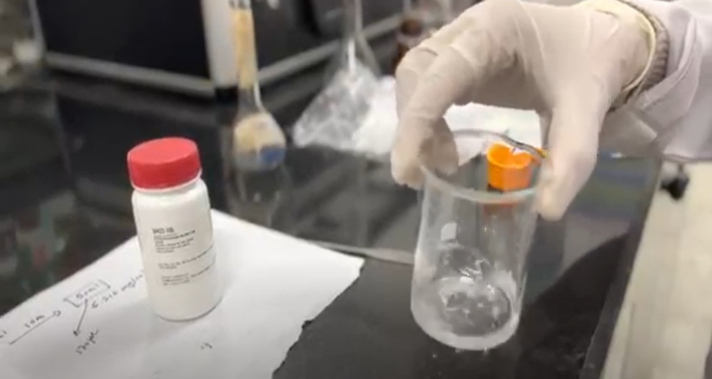


**C**

**Supplementary Figure S2. A.** Weigh β-Sodium Glycerophosphate accurately, **B.** Adding distilled water to the β-Sodium Glycerophosphate powder, **C.** Dissolving β-Sodium Glycerophosphate with distilled water.

1. **Preparation of 50 mg/L L-Ascorbic Acid Solution**

*L-ascorbic acid (formula: C_6_H_8_O_6_, molecular weight: 176.12 g/mol).*

**Step 1:** The required amount of L-ascorbic acid for a 50 mg/L solution was calculated:


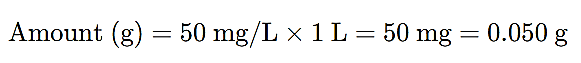


- 0.050 g (50 mg) of L-ascorbic acid powder was accurately weighed and dissolved in 50 mL of distilled water until fully dissolved.

**Step 2:** To achieve the same concentration in 510 mL, the required amount for 510 mL was calculated:


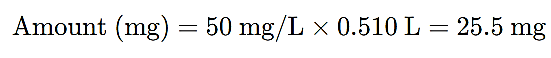


- Accurately weigh 25.5 mg of L-Ascorbic Acid powder, **Figure (3, A).**

**Step 3**: Dissolve the 25.5 mg of L-Ascorbic Acid in 510 mL of distilled water until fully dissolved, **Figure (3, B).** This ensures the final solution has the correct concentration of L-Ascorbic Acid in both 50 mL and 510 mL volumes.


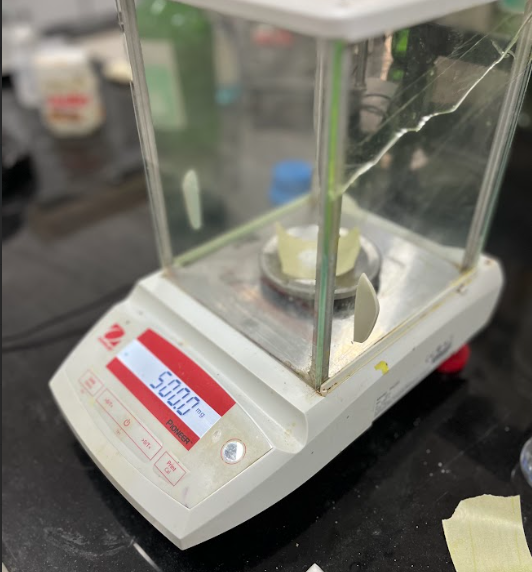


**A**


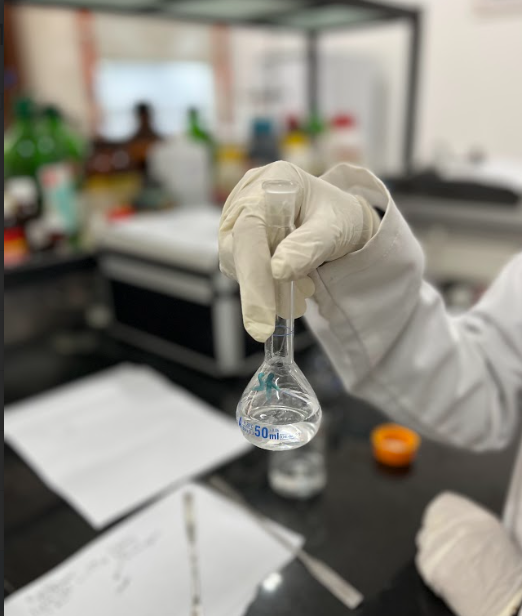


**B**

**Supplementary Figure S3.** Ascorbic acid was weighted **(A)** and dissolved with distilled water **(B).**

1. **Combining the Solutions**

*The final combined solution was prepared:*

- The solutions of dexamethasone, L-ascorbic acid, and β-sodium glycerophosphate were mixed, ensuring the final combined volume was 510 mL, **Figure (4).**
- This involved using the previously prepared solutions and adding distilled water as needed to ensure the final volume remained 510 mL.
- By following these steps, the correct final concentrations of dexamethasone (10⁻⁸ mol/L), L-ascorbic acid (50 mg/L), and β-sodium glycerophosphate (10 mmol/L) in the final 510 mL solution were achieved. Each component was prepared and then combined carefully to maintain the overall volume and concentration.


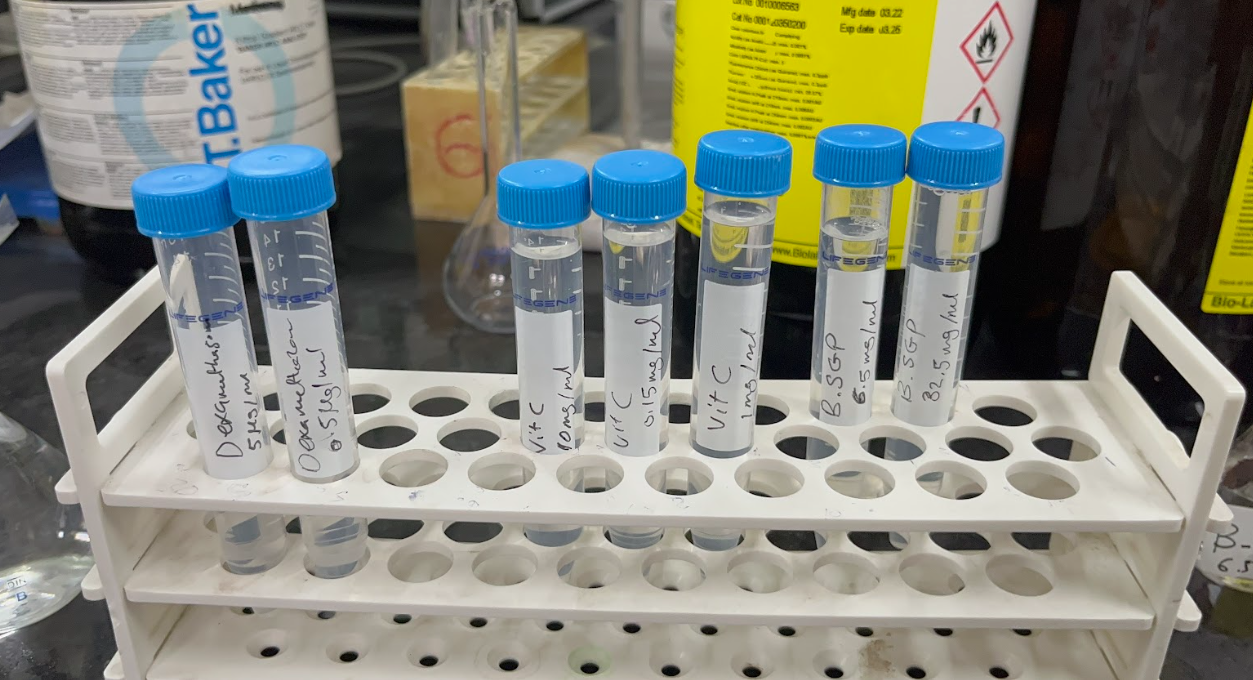


**Supplementary Figure S4.** Stock solutions of dexamethasone, β-sodium glycerophosphate and ascorbic acid.


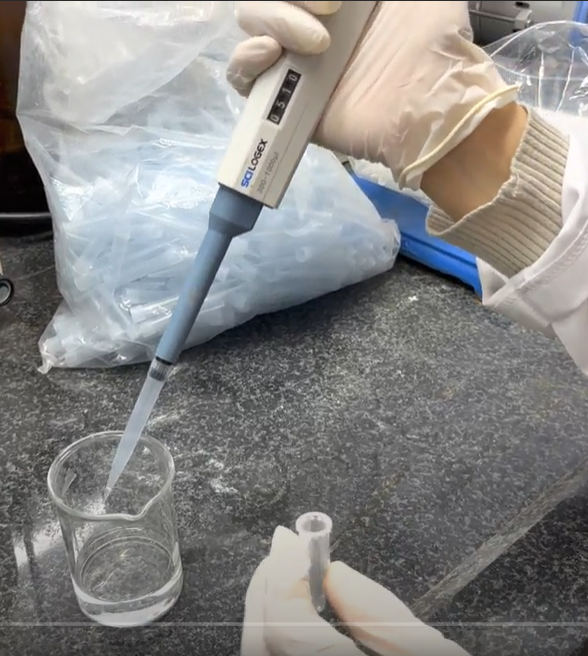

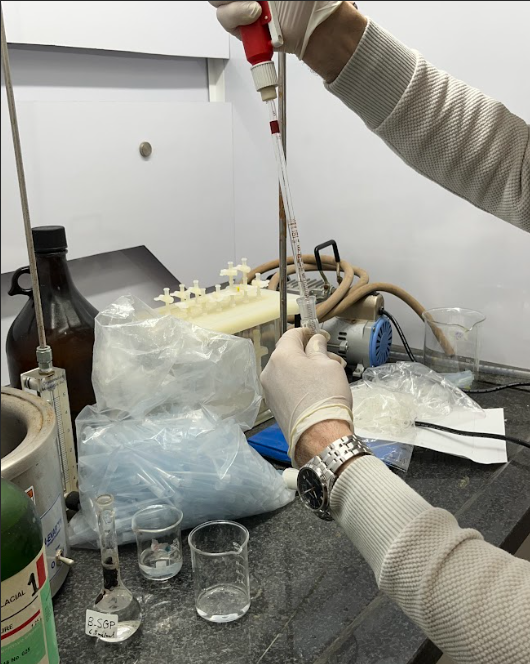


**B**

**A**

**Supplementary Figure S5. A** and **B** Pipettes used to measure and aliquot 510 mL of the combined solutions.

1. **The Combined Solution Storage**

- The combined solution was sterilized using a sterile 0.22-micron filter. It is one common and frequently used technique for eliminating microorganisms from heat-sensitive solutions **(Hahn, 2004)**.
- The sterilized solution was aliquoted into sterile containers and stored at (-20 to -55°C) for long-term storage, **Figures (5)** and **(6).**


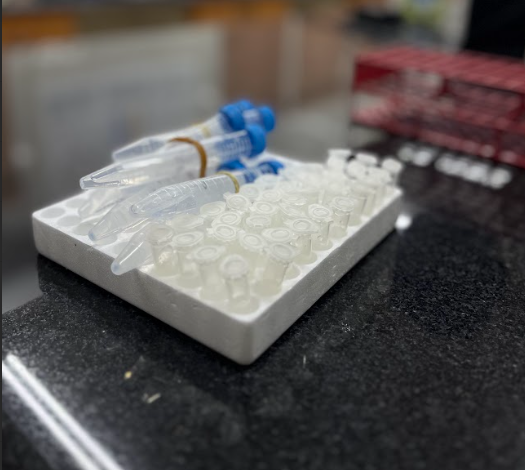

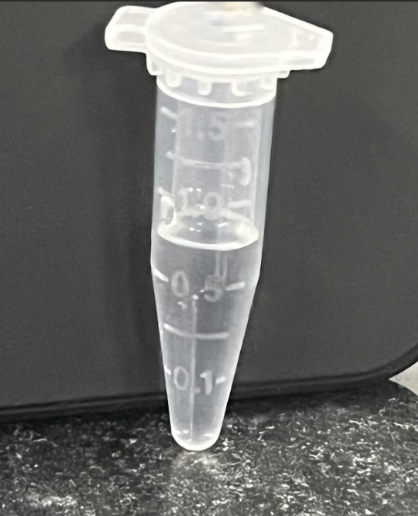


**Supplementary Figure S6.** The aliquoted osteogenic inducer, divided according to the maximum absorption capacity of the gelatin sponge per case.


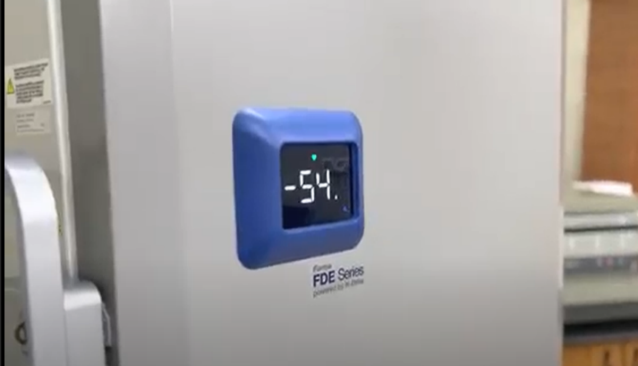


**A**


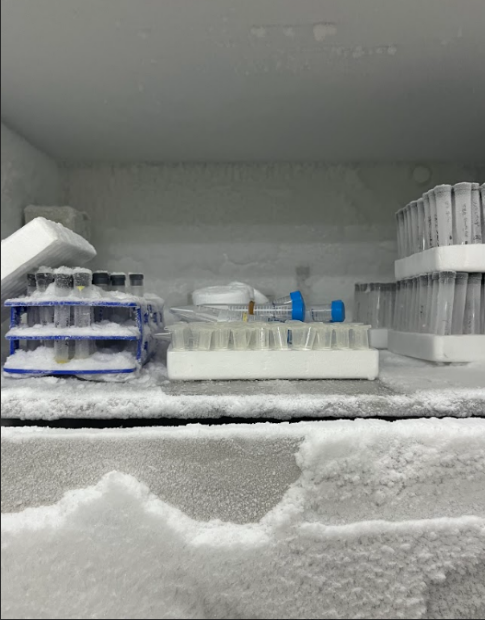


**B**

**Supplementary Figure S7.** Storage at (-20 to -55°C) freezer.

1. **Sponge Absorption**

- A 5 mm x 15 mm x 10 mm gelatin sponge (Jinling Pharmaceutical Company, Nanjing, China) was soaked in the prepared osteogenic inducer solution, ensuring it absorbed approximately 75 mg of the solution.
- The absorption was verified by weighing the sponge before and after soaking.
- Post-absorption, the sponge was checked to ensure it retained the solution without significant loss.
- This procedure ensured that the concentrations of drugs were maintained when absorbed by the gelatin sponge, meeting the study’s requirements for drug delivery and implant osseointegration

***Supplementary Material 3: Study Hypotheses***

Study hypothesis illustrating the null (H₀) and alternative (H₁) hypotheses, grouping of implants (test and control), and outcome interpretation based on implant stability measurements (ISQ).


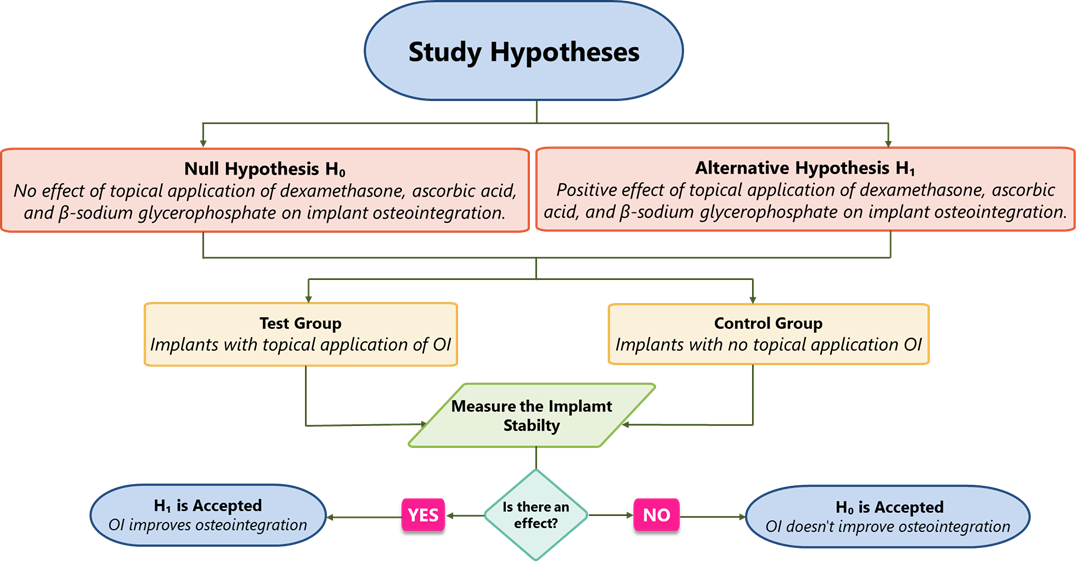


**Supplementary Figure S8. Study hypothesis flowchart showing the null (H₀) and alternative (H₁) hypotheses, the allocation of implants into test and control groups, and the decision process based on ISQ outcomes.**

***Supplementary Material 4: Detailed follow-up schedule for each implant***

**Supplementary Table S1. ISQ measurements of the test implants during the 16-week follow-up period (January – September 2023).**

| Period  ID | Baseline | | 4 weeks | | 8 weeks | | 16 weeks | |
| --- | --- | --- | --- | --- | --- | --- | --- | --- |
|  | **Test** | **Control** | **Test** | **Control** | **Test** | **Control** | **Test** | **Control** |
| 1 | 77 | 67 | 75 | 71 | 73 | 68 | 81 | 80 |
| 2 | 81 | 69 | 64 | 63 | 70 | 68 | 66 | 73 |
| 3 | 64 | 68 | 75 | 62 | 68 | 75 | 75 | 74 |
| 4 | 60 | 66 | 83 | 63 | 73 | 75 | 68 | 68 |
| 5 | 64 | 70 | 79 | 67 | 70 | 67 | 73 | 68 |
| 10 | 68 | 68 | 70 | 68 | 59 | 60 | 70 | 68 |
| 11 | 60 | 75 | 68 | 61 | 74 | 73 | 68 | 75 |
| 12 | 73 | 69 | 70 | 63 | 75 | 73 | 78 | 77 |
| 13 | 61 | 62 | 66 | 63 | 79 | 66 | 79 | 79 |
| 14 | 70 | 62 | 68 | 68 | 76 | 80 | 71 | 69 |
| 15 | 49 | 58 | 66 | 73 | 69 | 61 | 74 | 73 |
| 16 | 59 | 77 | 60 | 58 | 70 | 65 | 80 | 82 |
| 17 | 73 | 75 | 68 | 68 | 66 | 63 | 76 | 77 |
| 18 | 57 | 61 | 43 | 49 | - | - | - | - |

Minor variations in follow-up timing (±3 days) were accepted due to patient scheduling constraints. All implants were included in the statistical analysis according to the planned time intervals (baseline, 4, 8, and 16 weeks). One paired set of implants (test and control) failed before completing the 16-week follow-up. All available ISQ readings for both implant up to the point of failure were included in the analysis.

***Supplementary Figure S9: Ethical Approval of Helsinki Committee of the Palestinian Health Research Council (PHRC)***


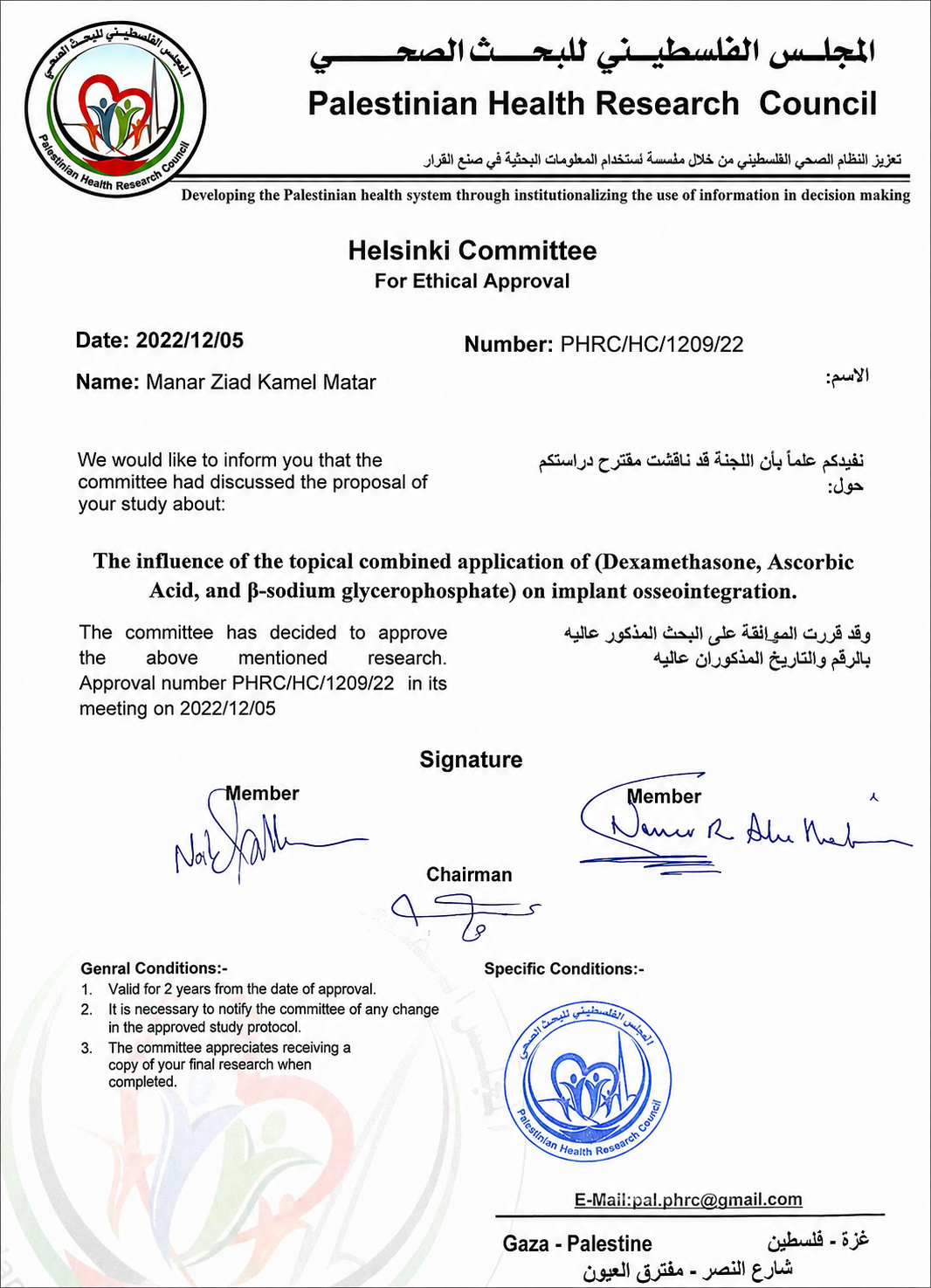


***Supplementary Figure S10: Approval of The Palestinian Medical Relief Society (PMRS)***


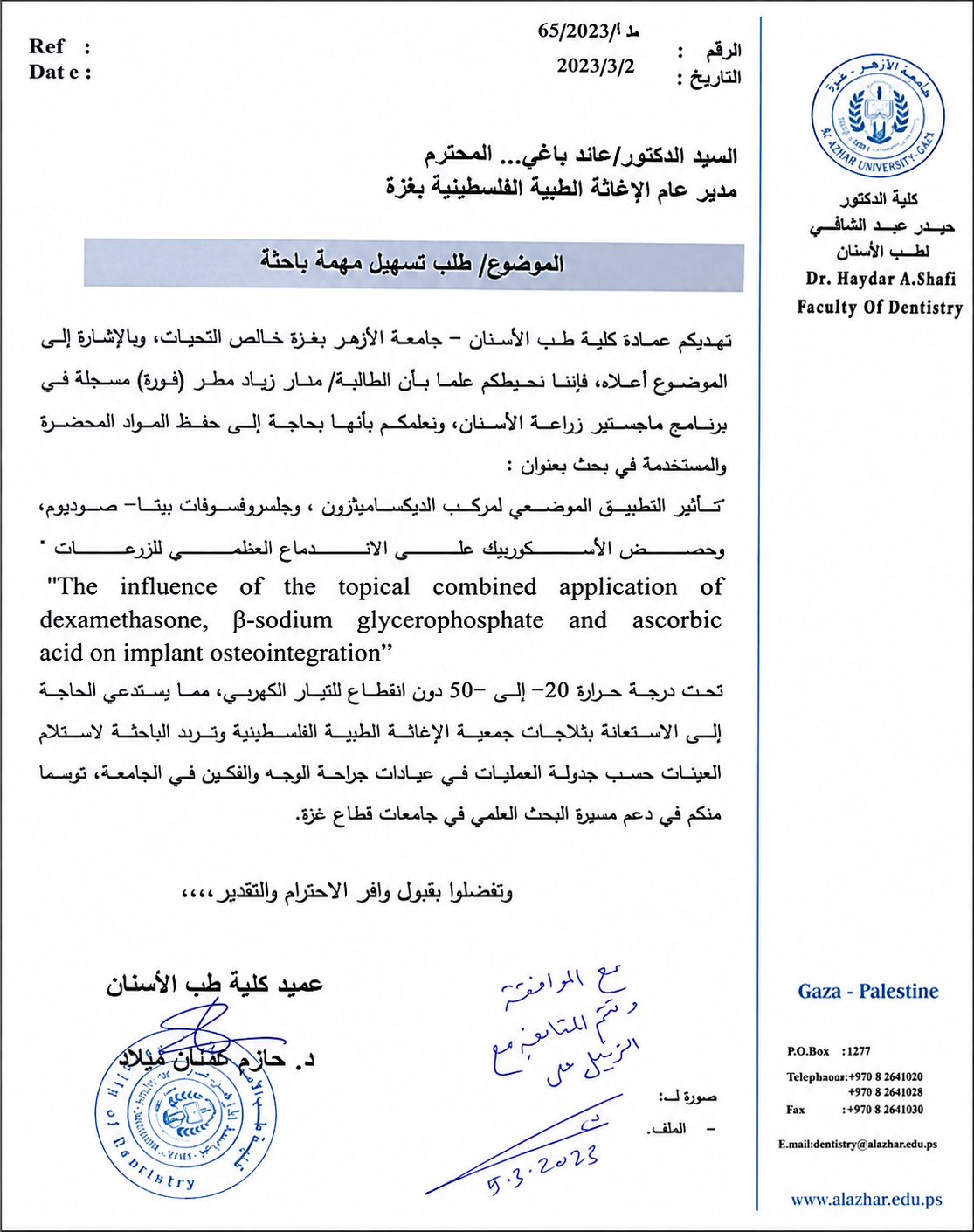


***Supplementary Form 1 – Informed Consent Form (Arabic)***


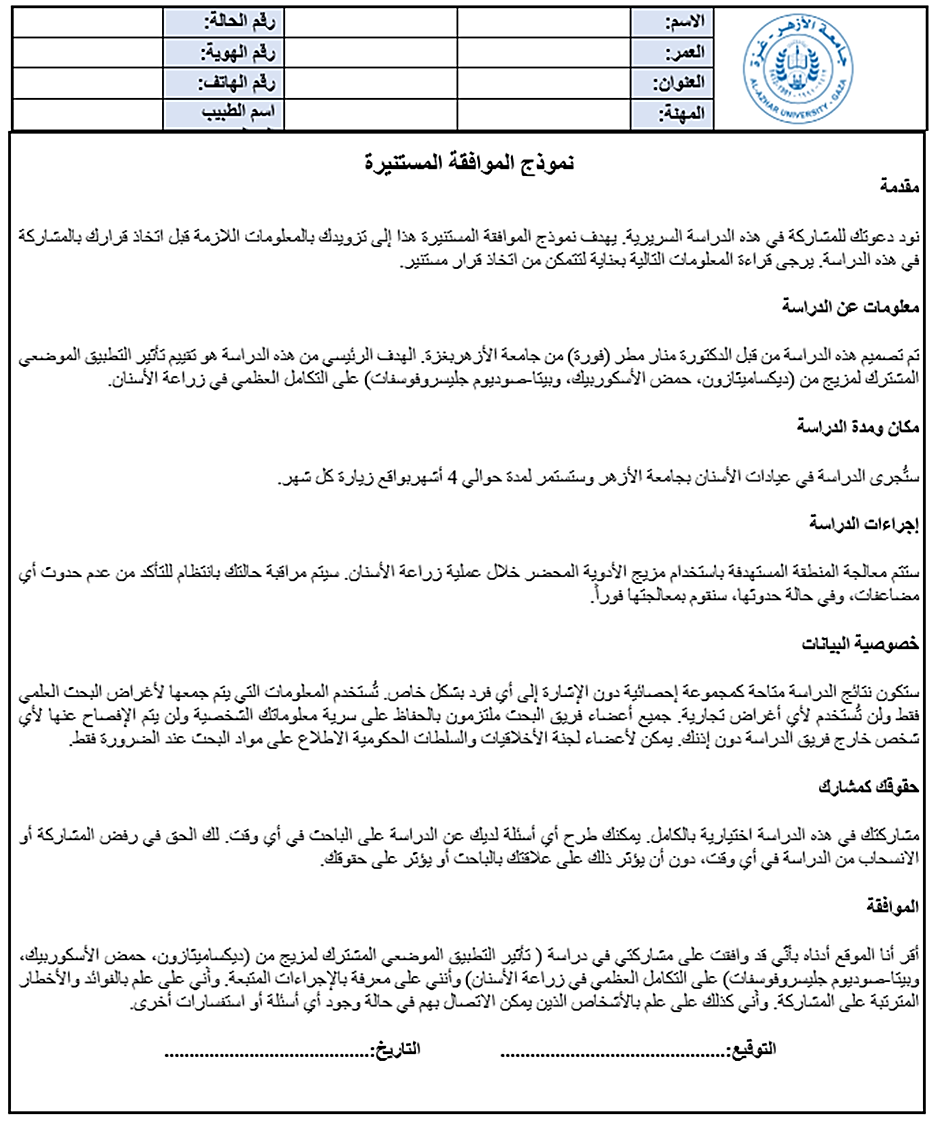


***Supplementary Form 2 – Informed Consent Form (English)***


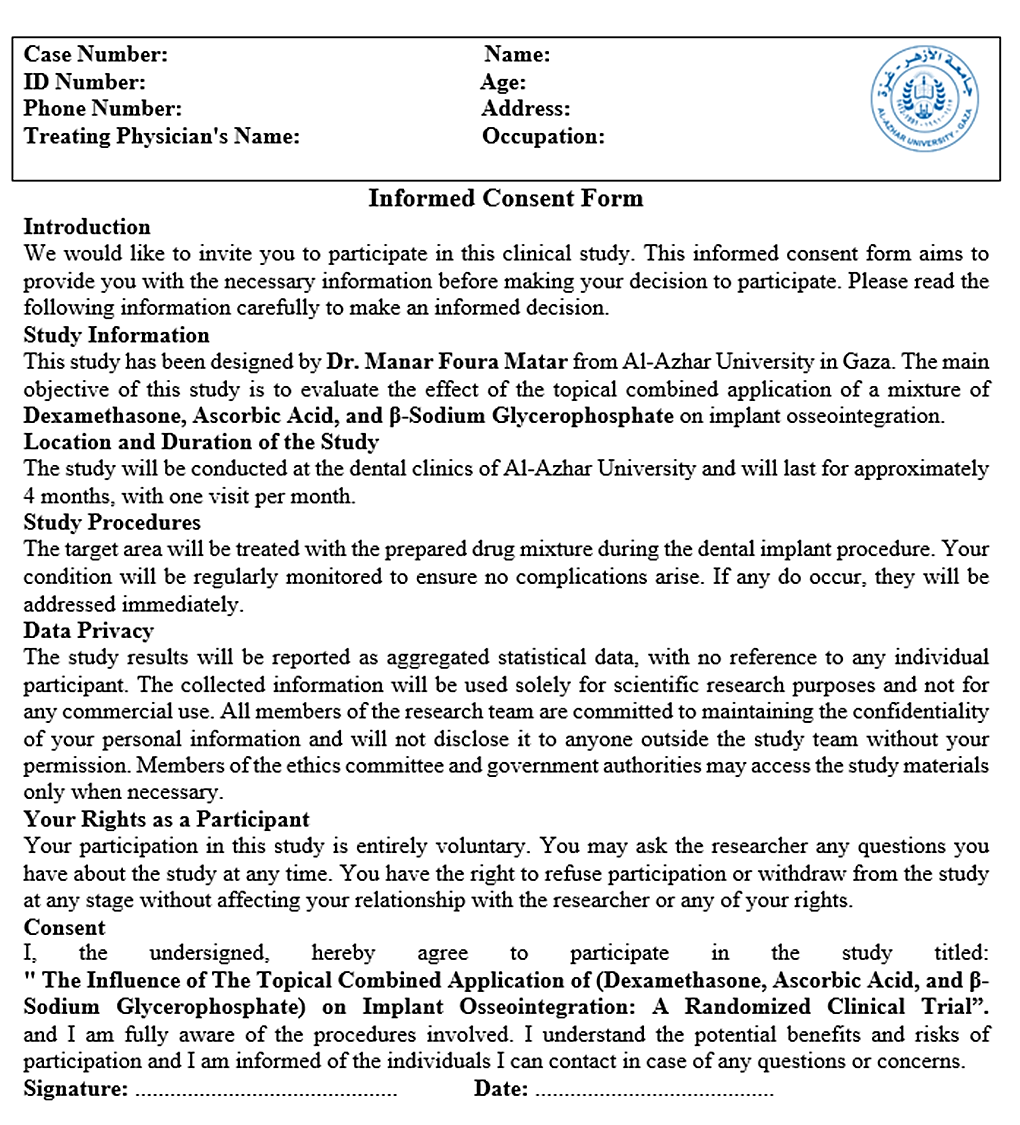


***Supplementary File 1 – Safety Data Sheet: Dexamethasone (D4902)***


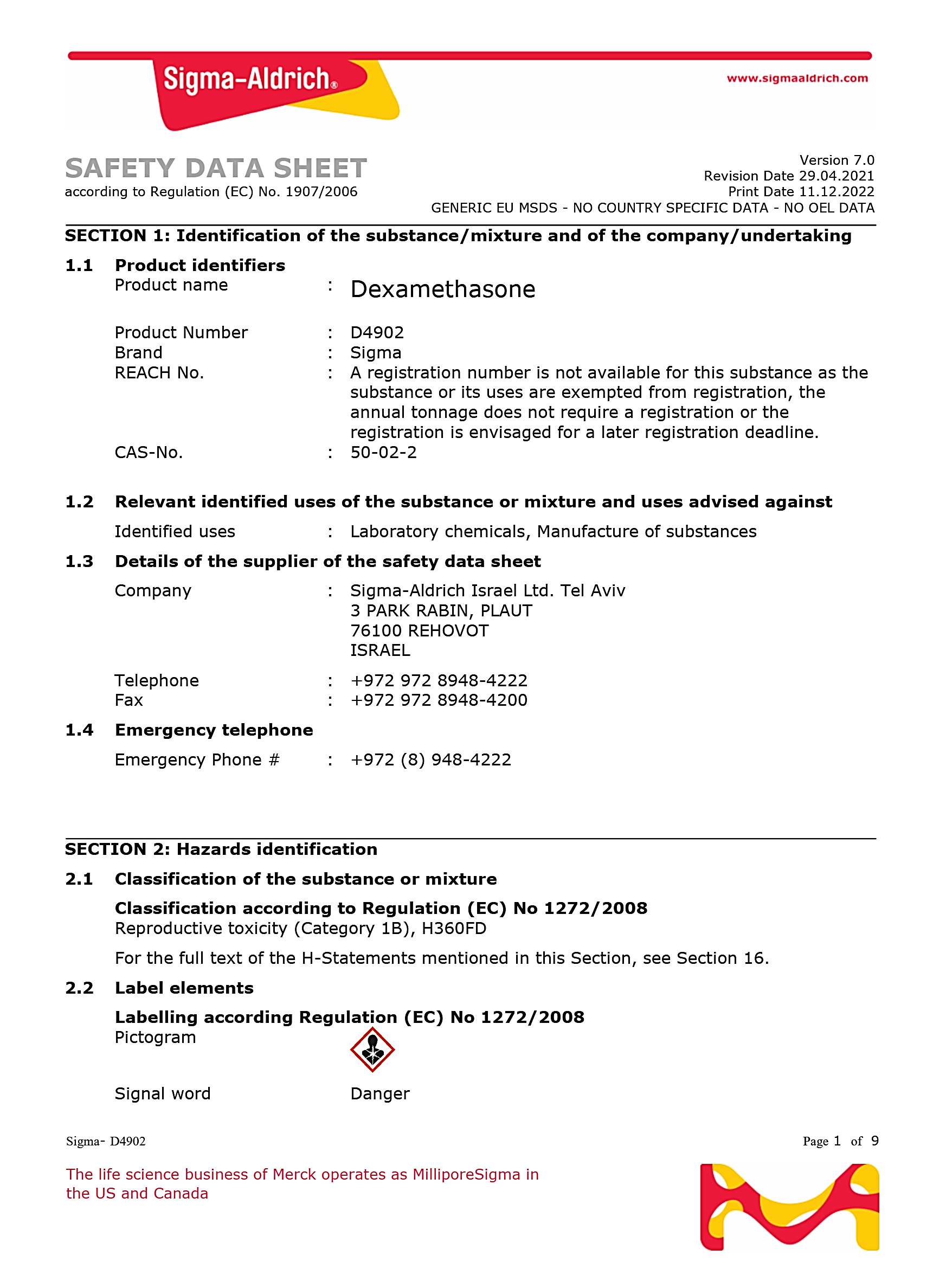


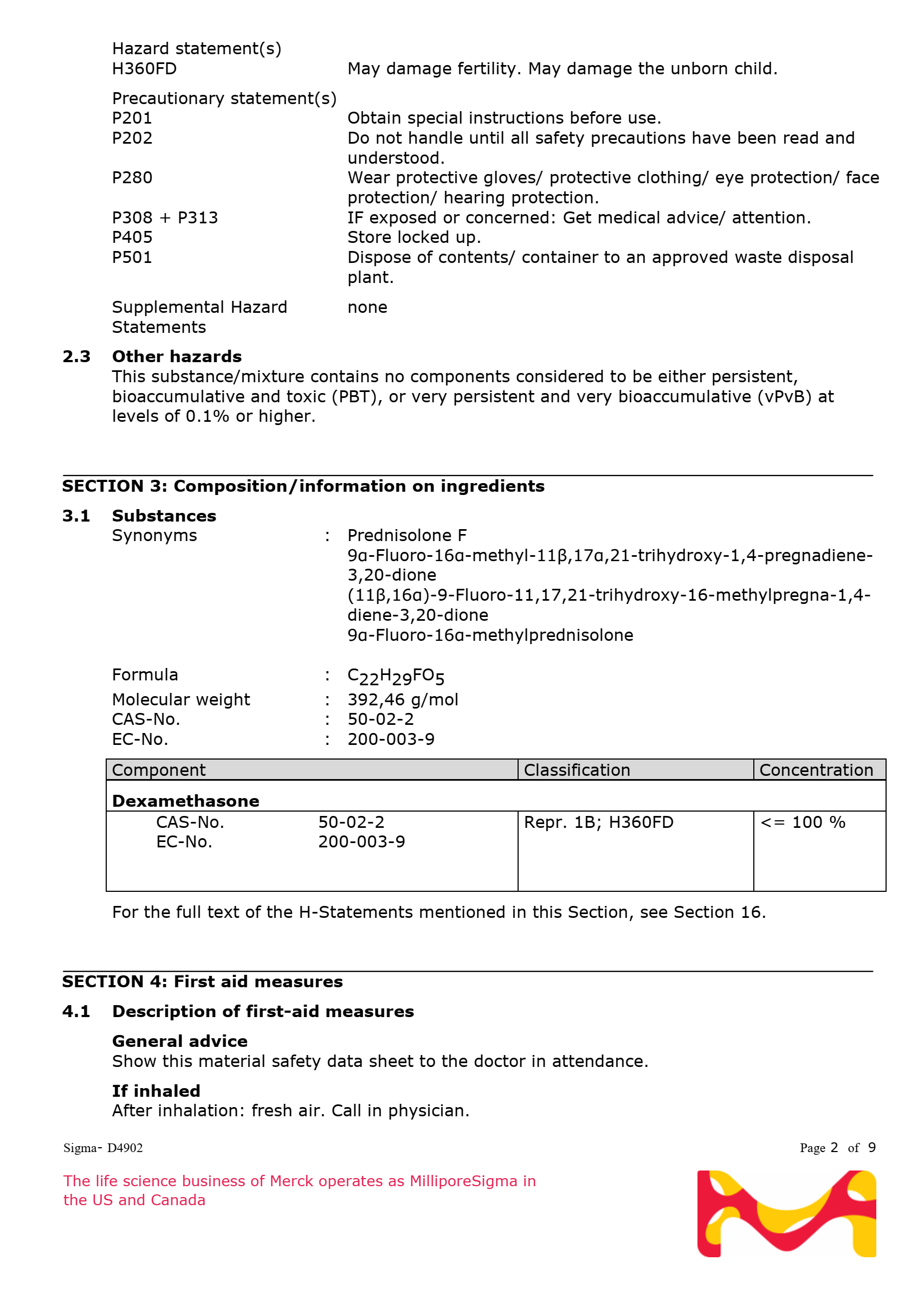


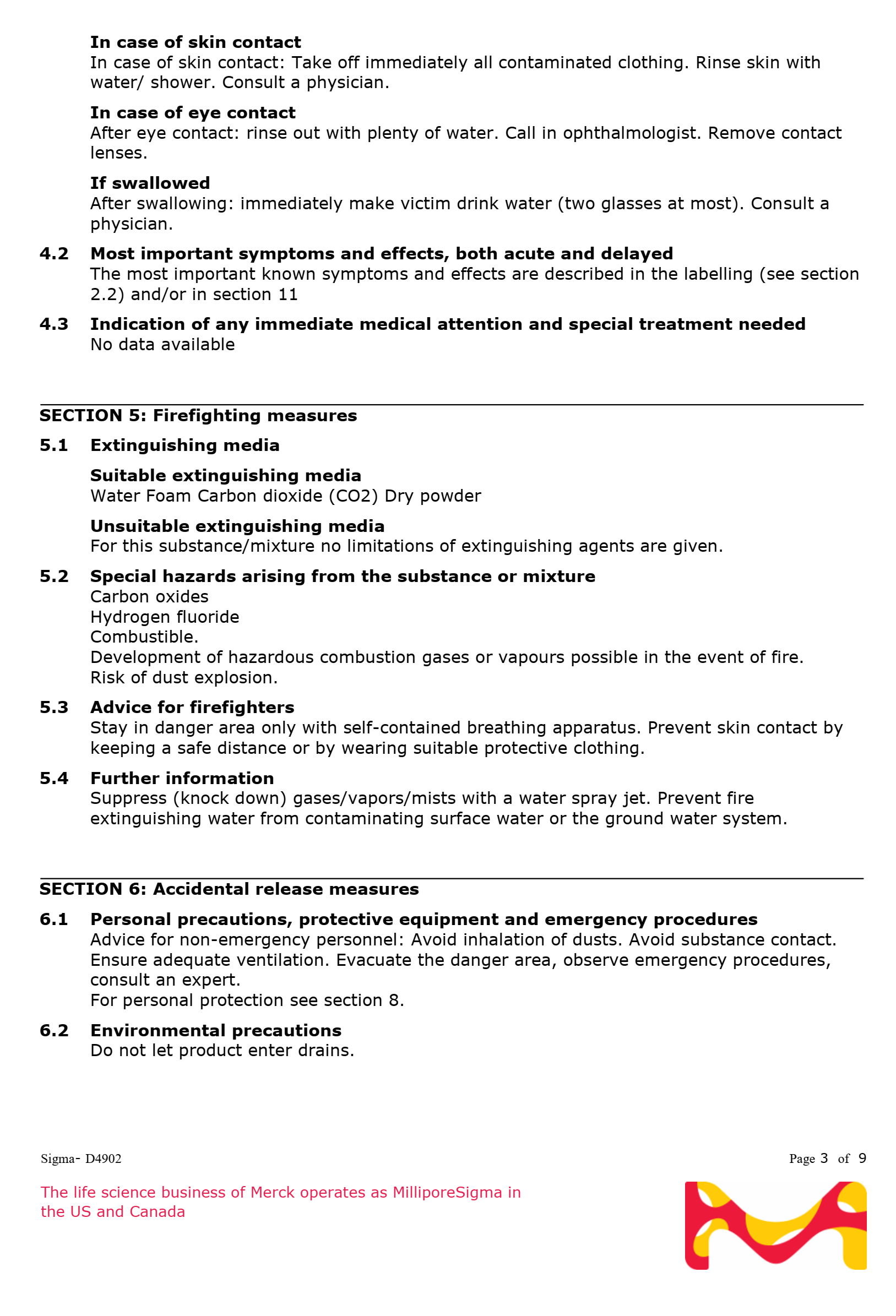


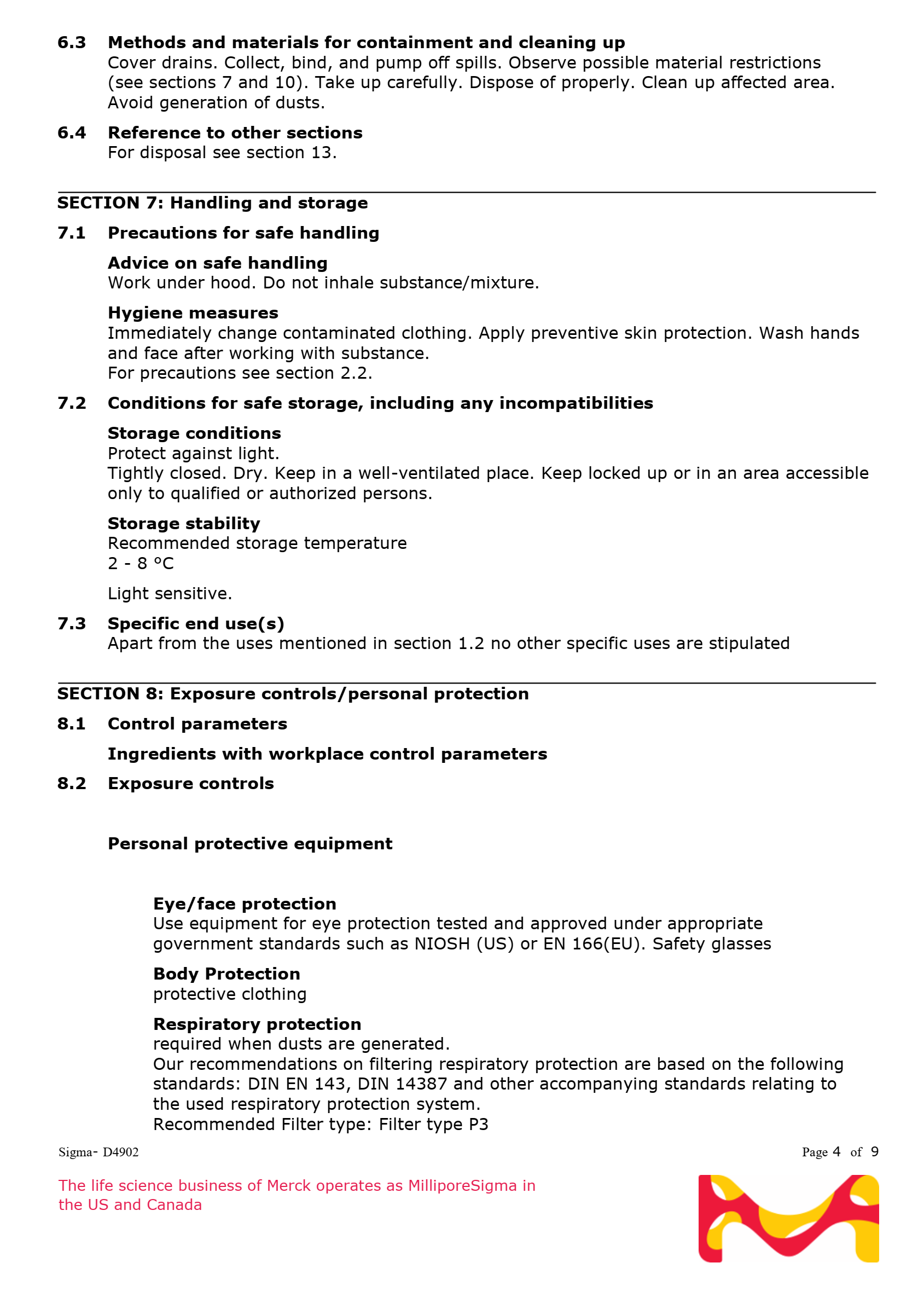


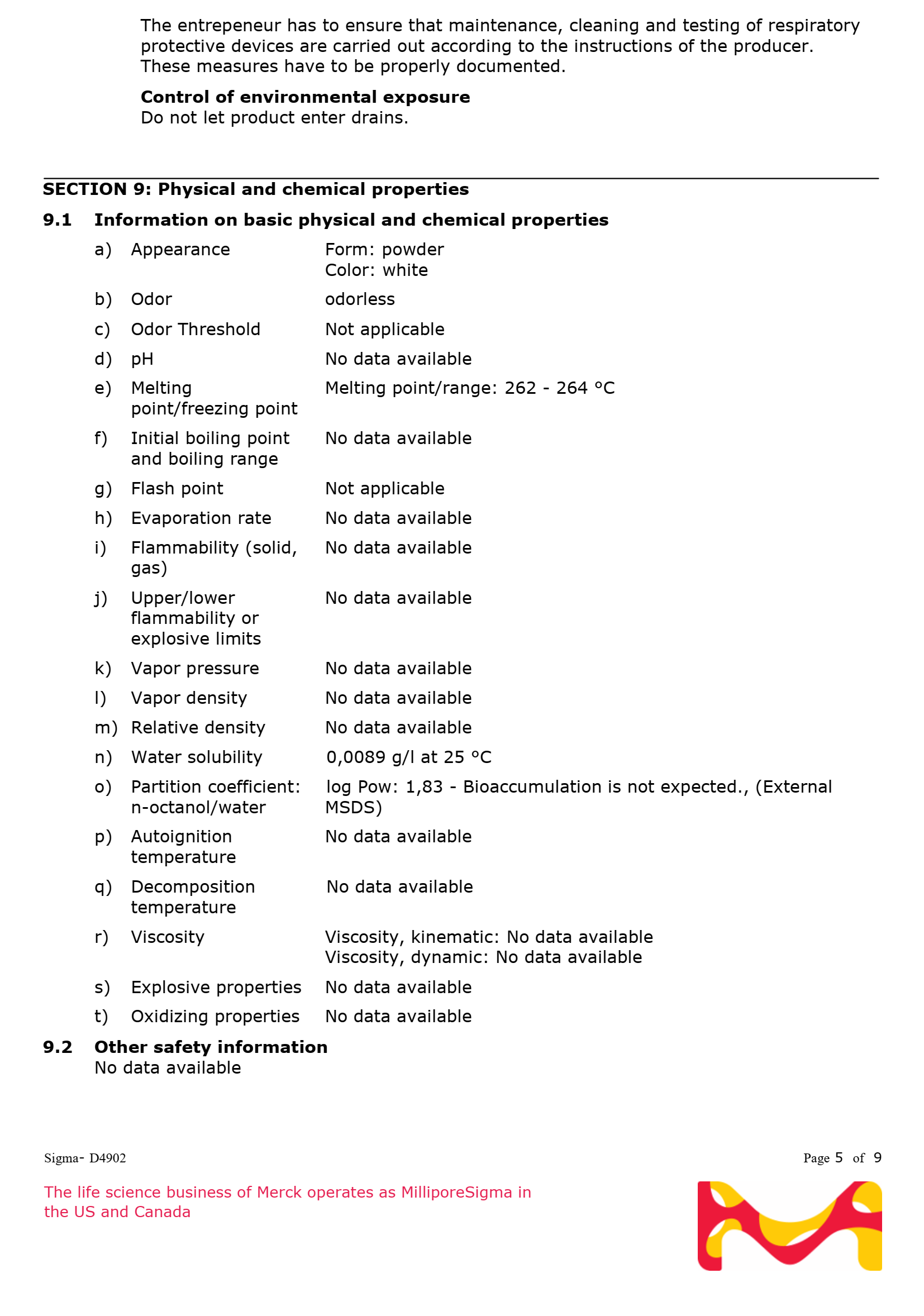


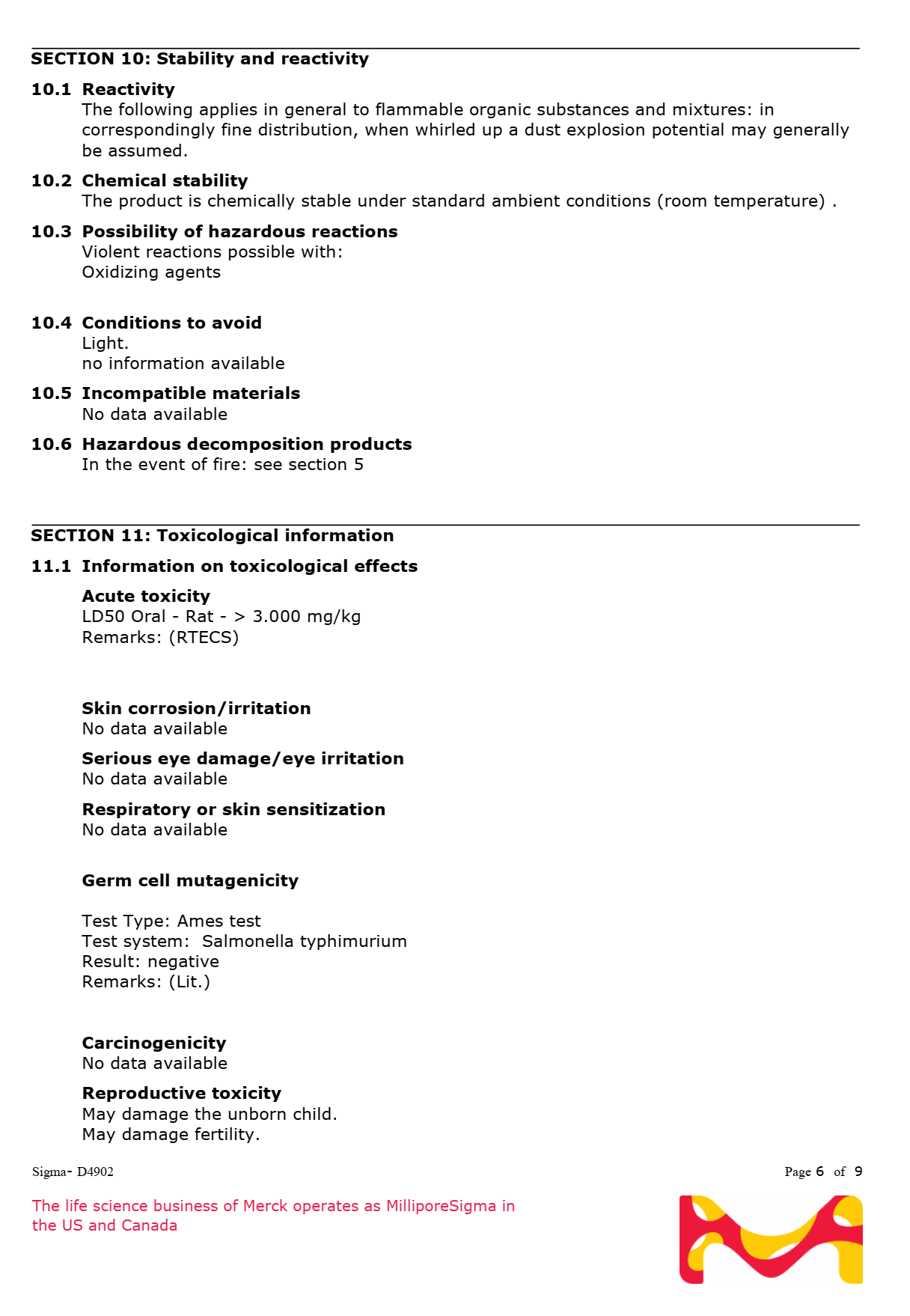


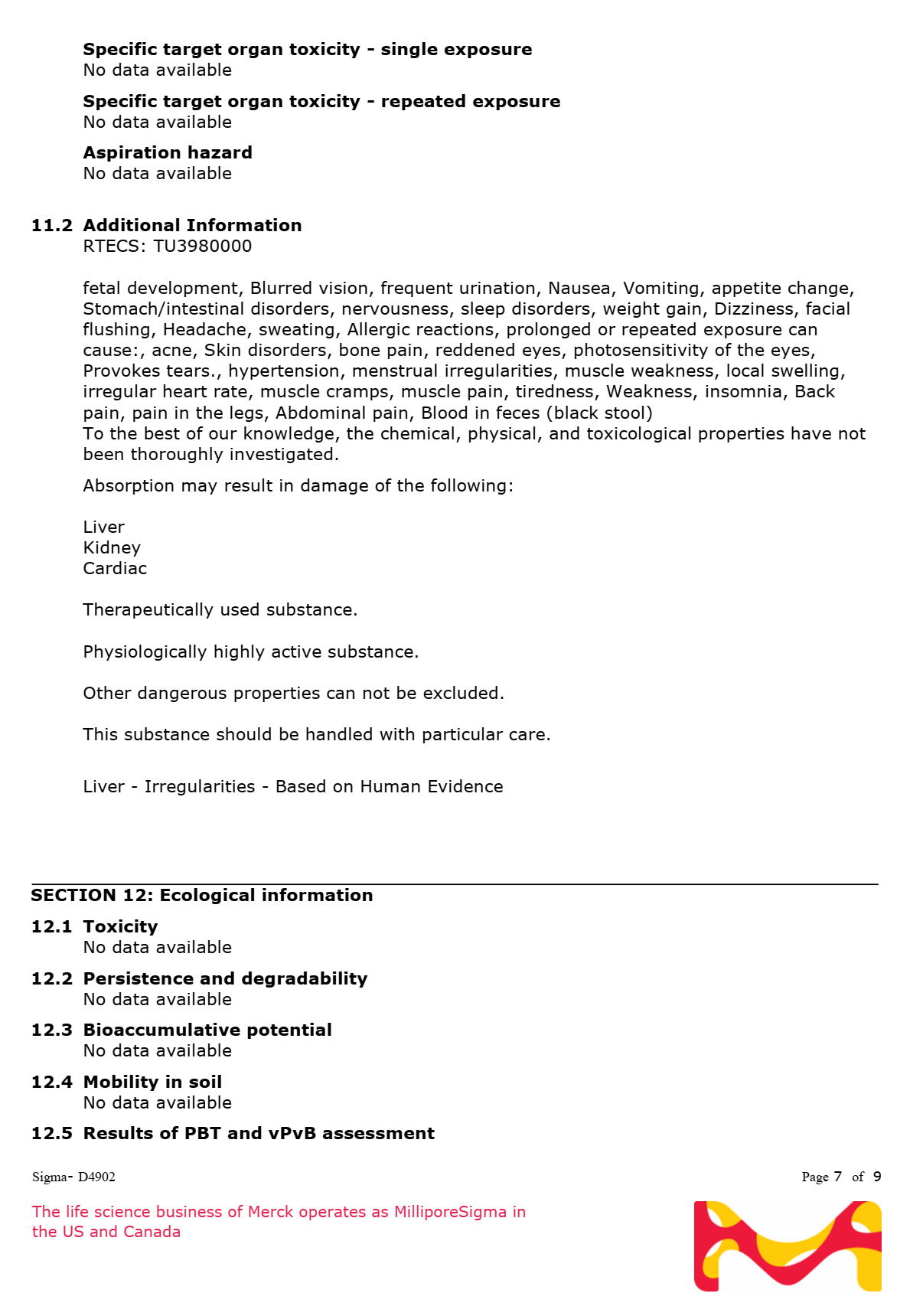


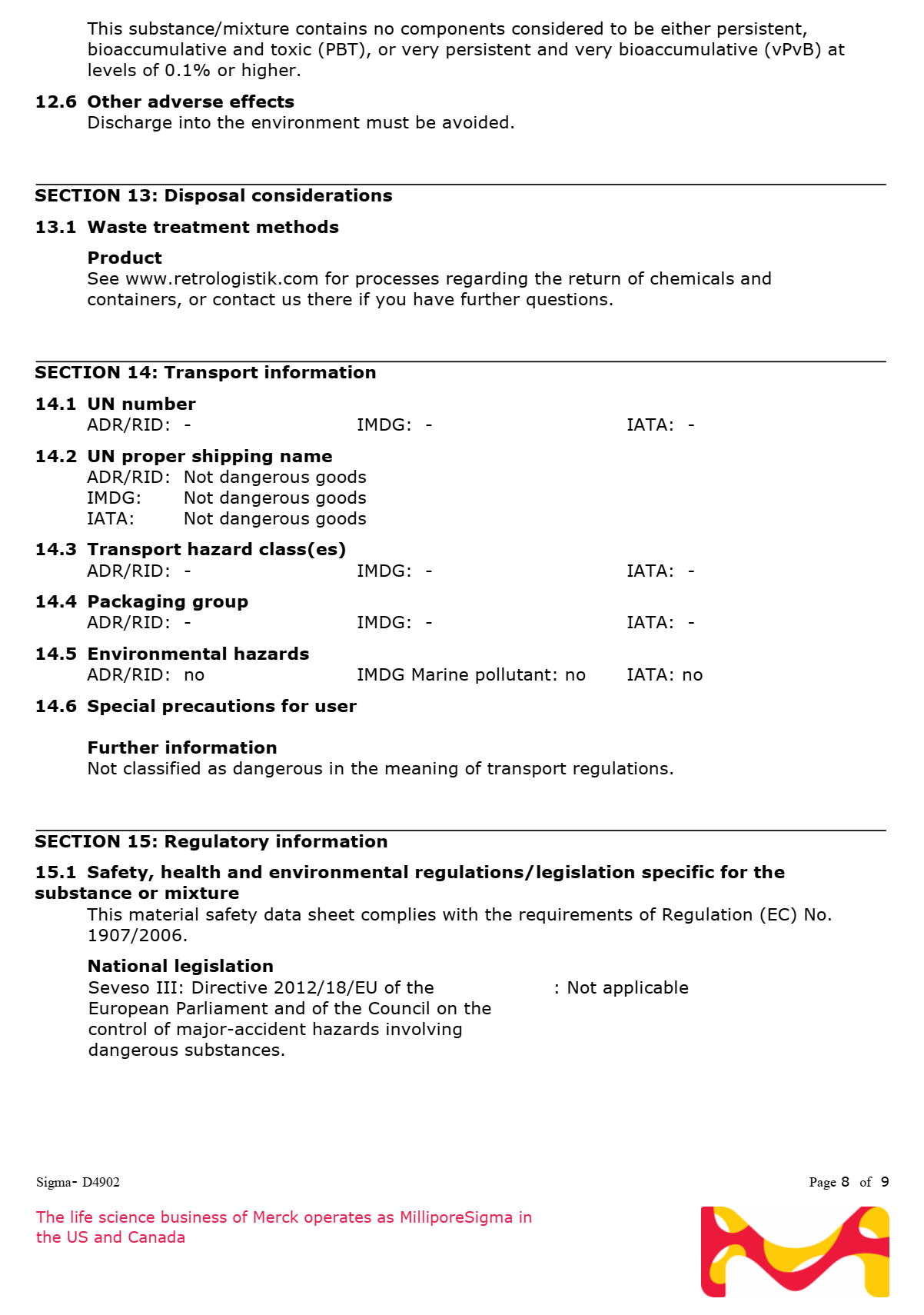


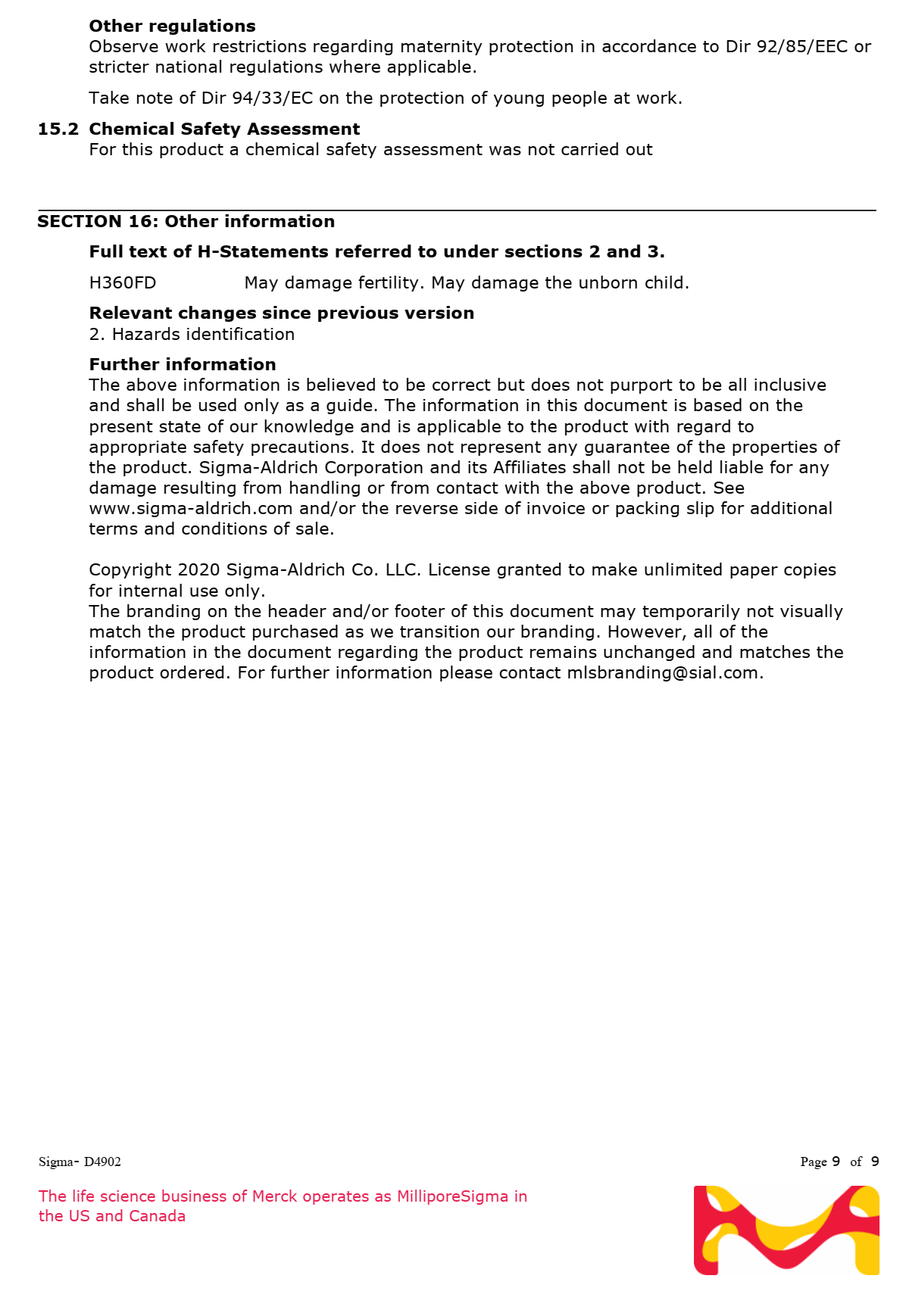


***Supplementary File 2 – Certificate of Analysis: Dexamethasone (Batch BCCJ0003)***


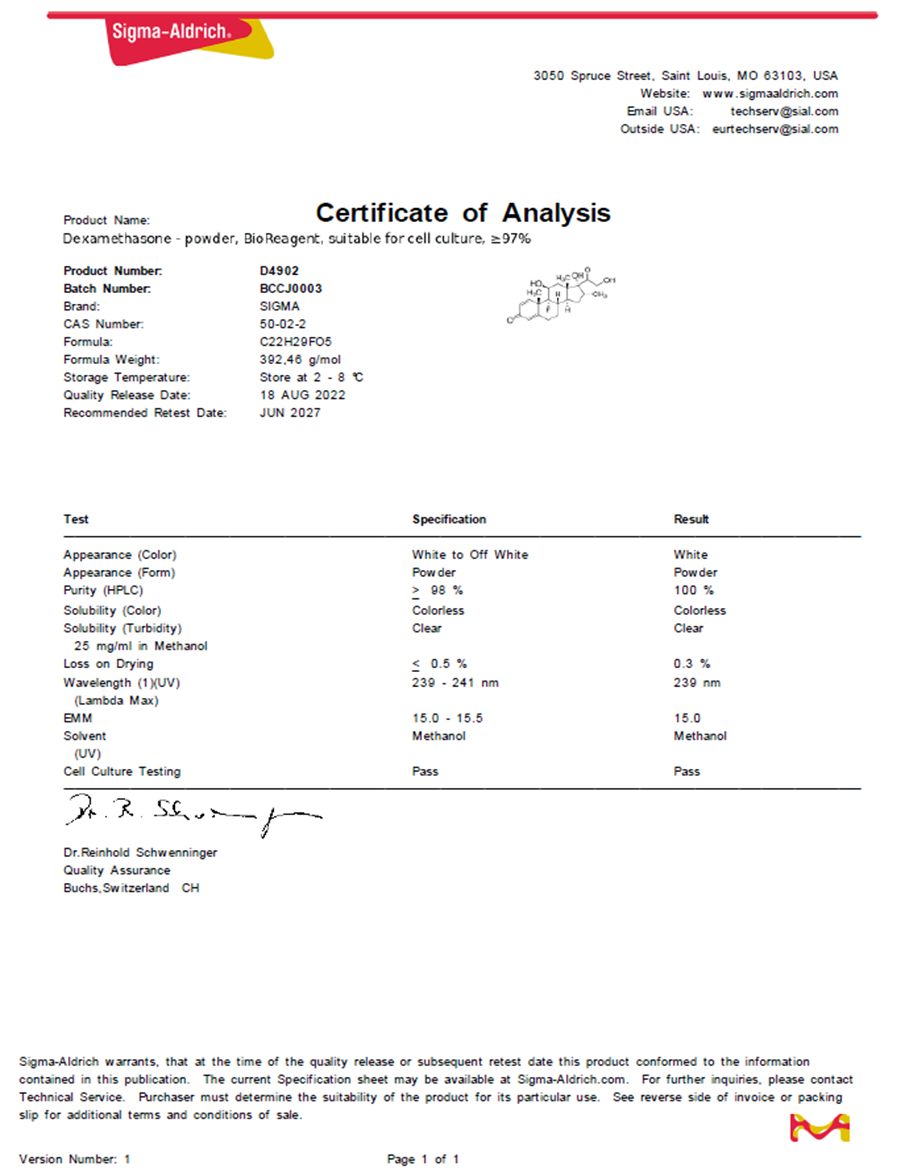


***Supplementary File 3 – Safety Data Sheet: β-Glycerophosphate disodium salt hydrate (G9422)***


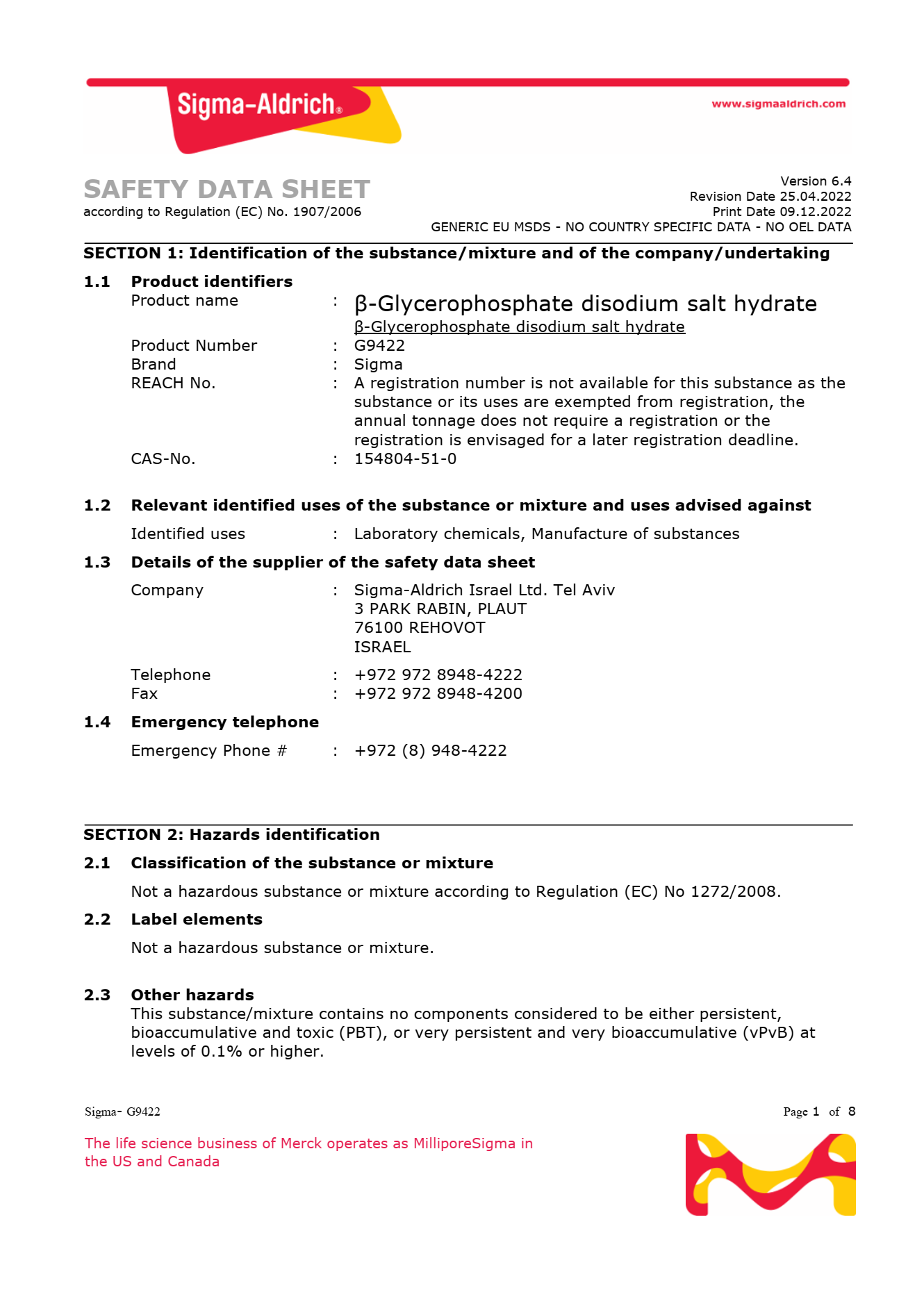


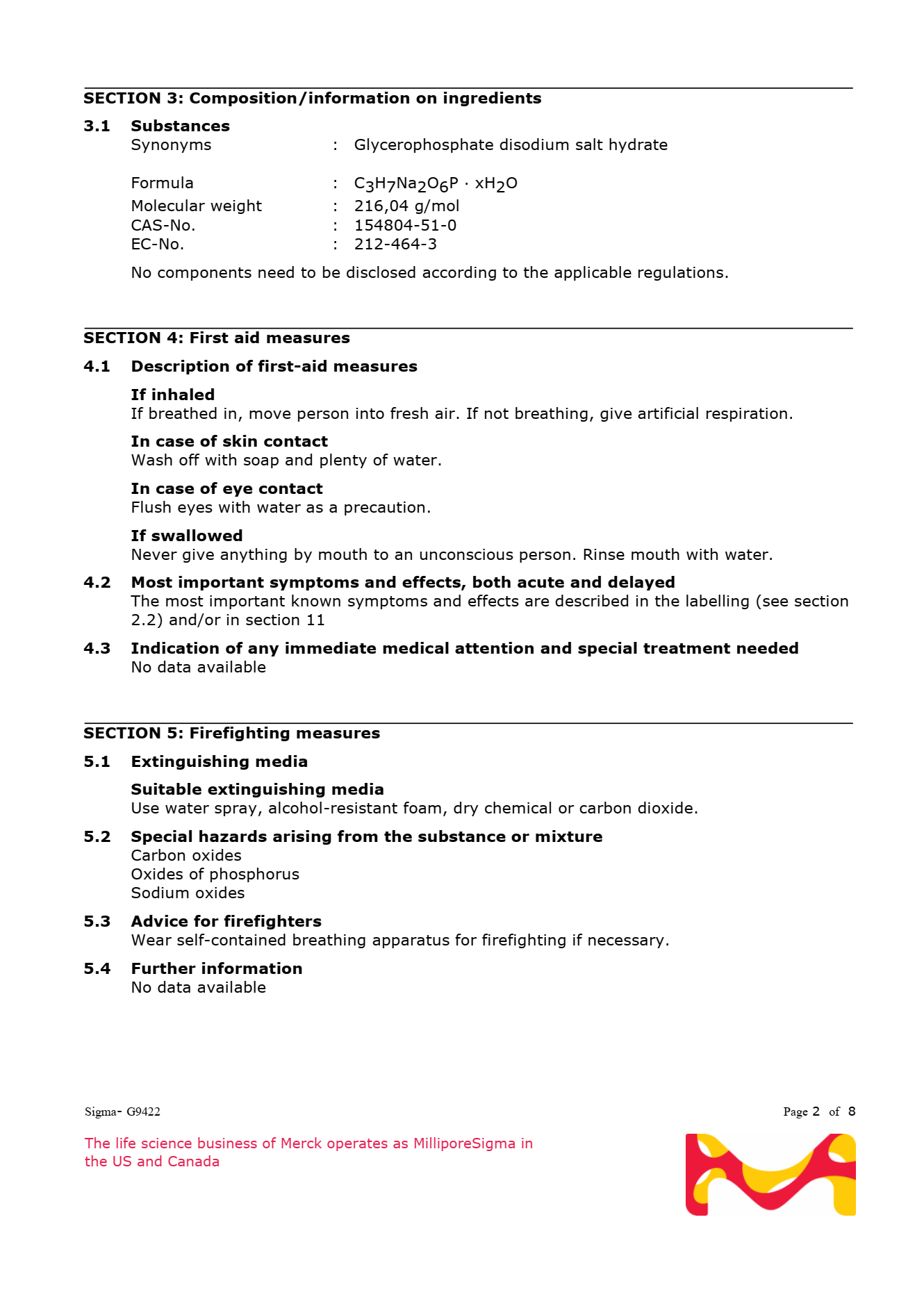


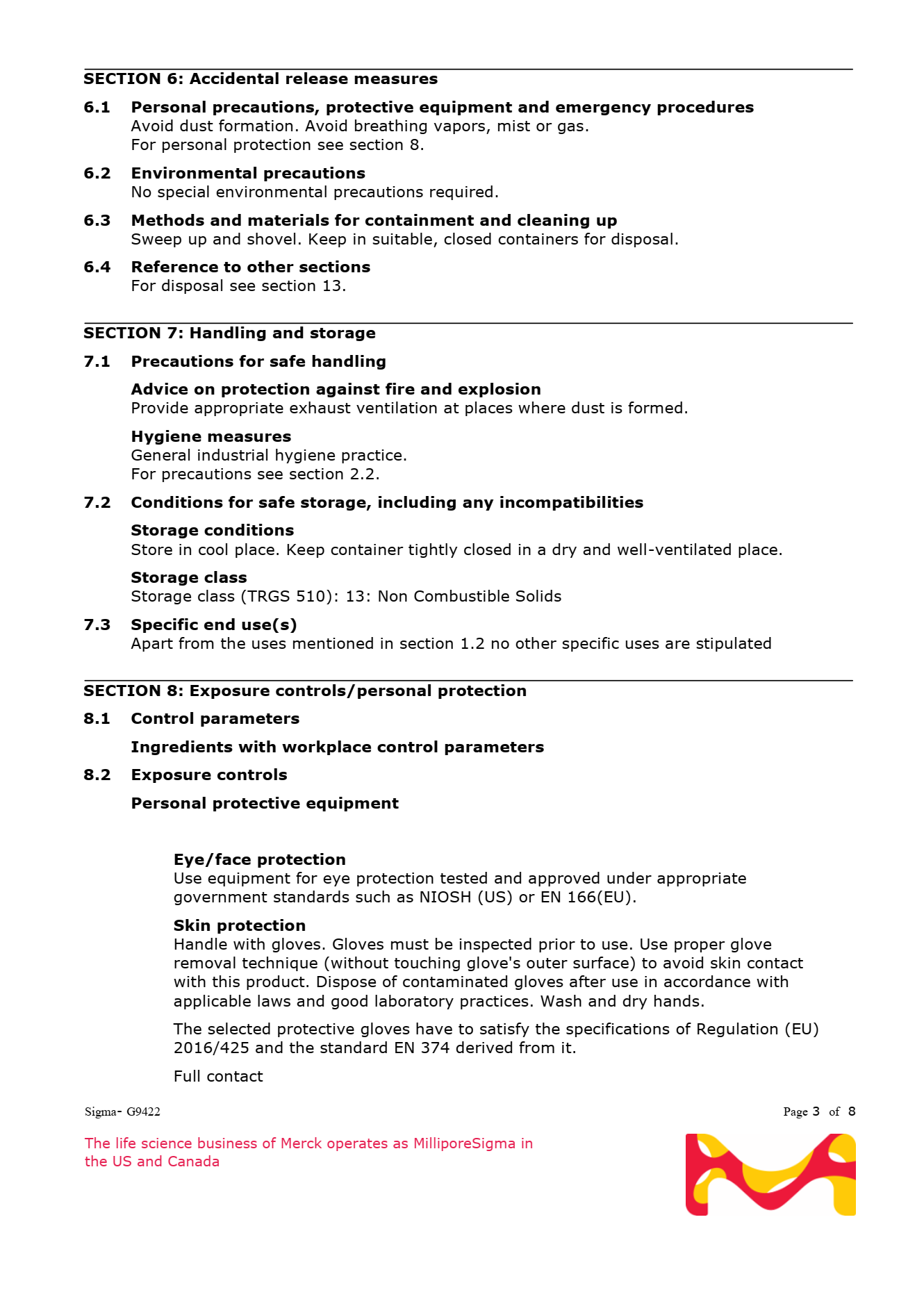


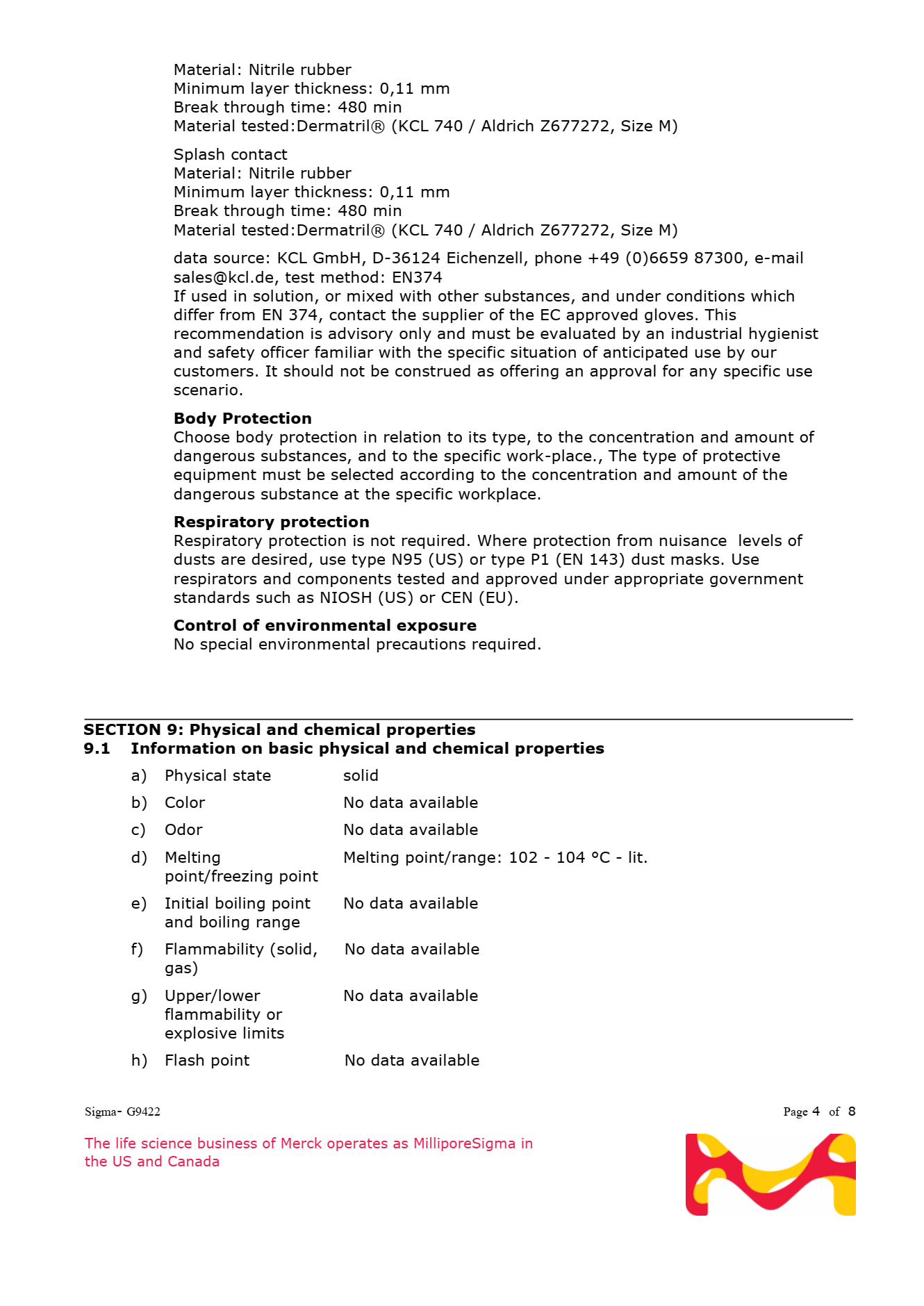


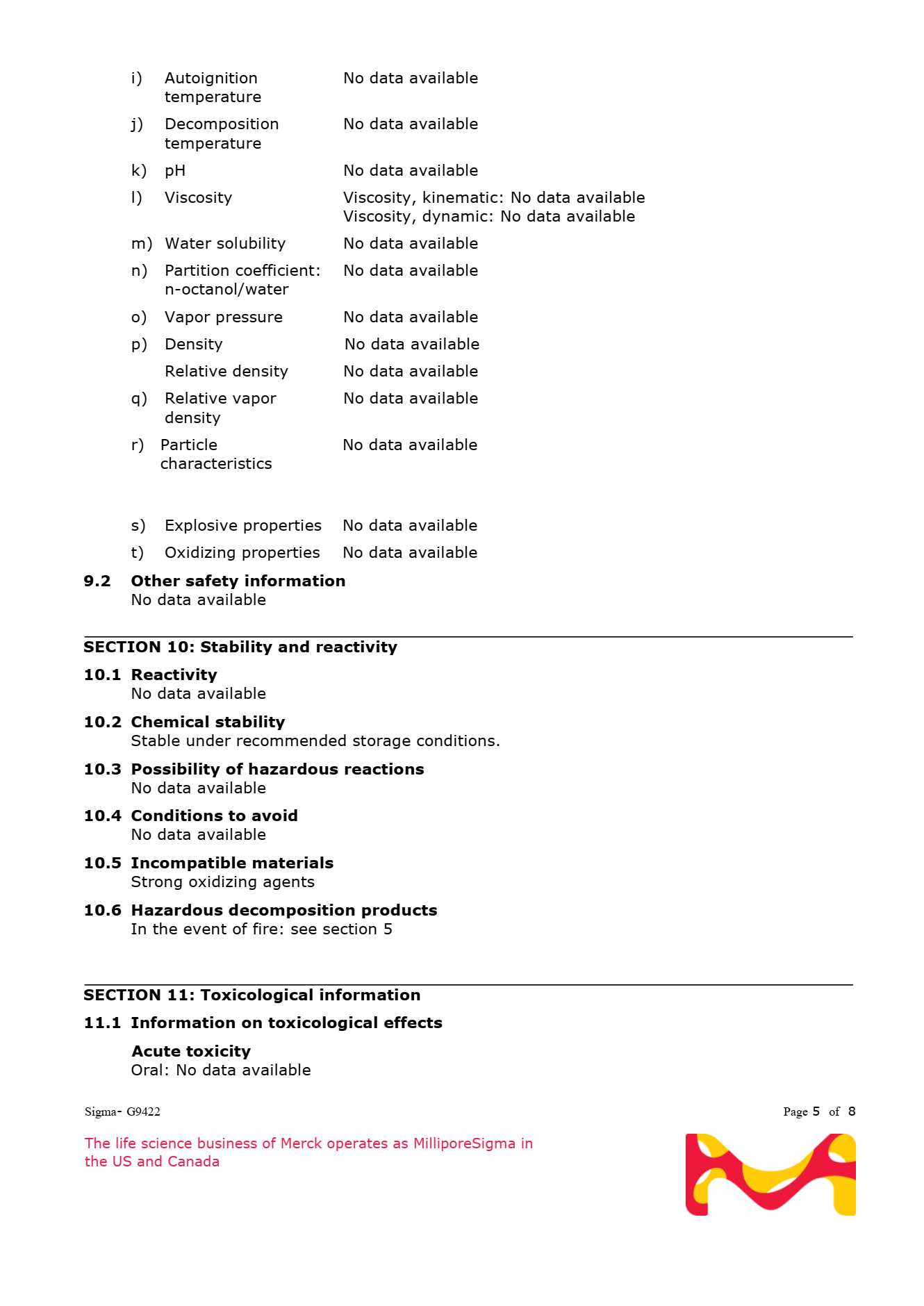


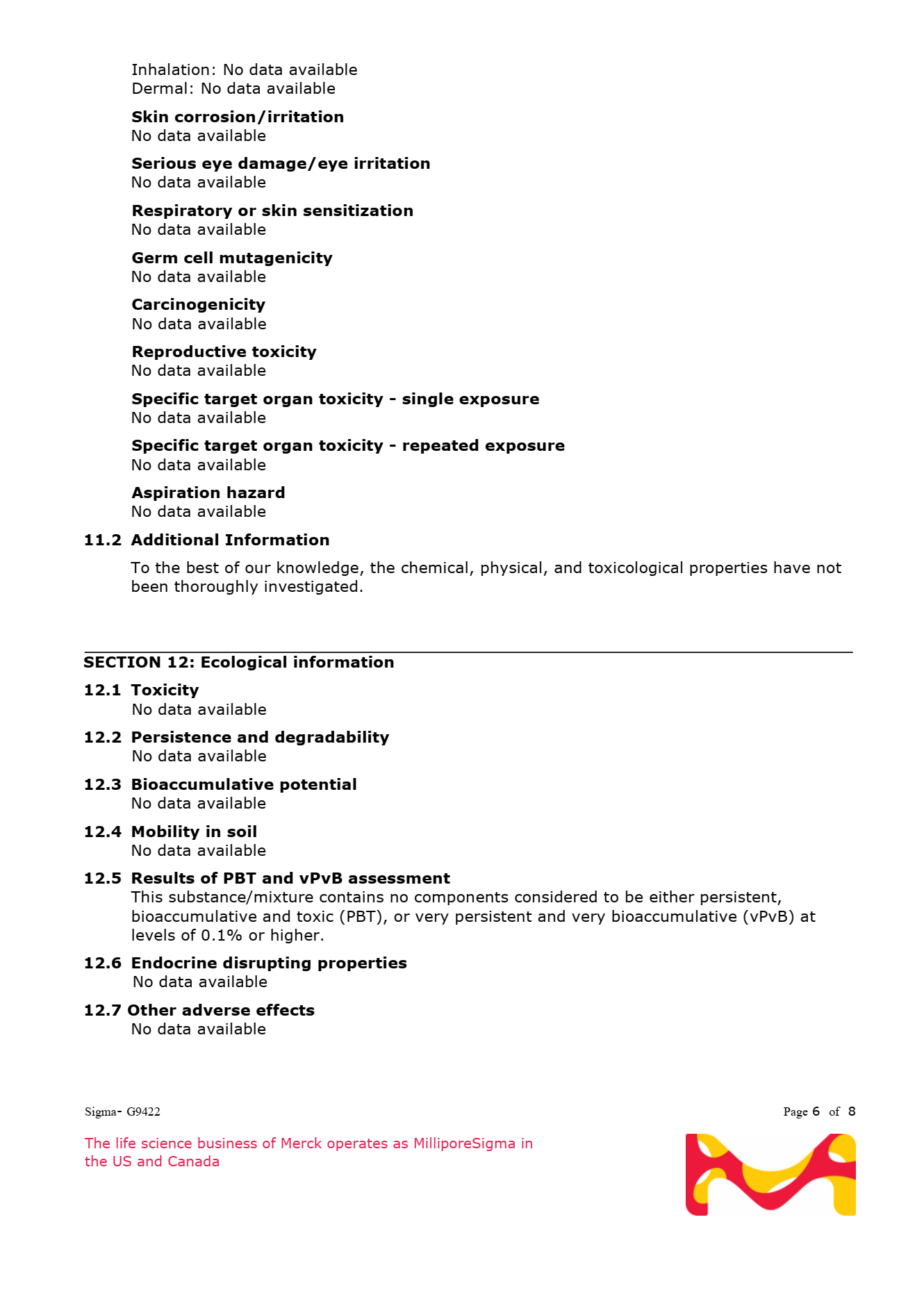


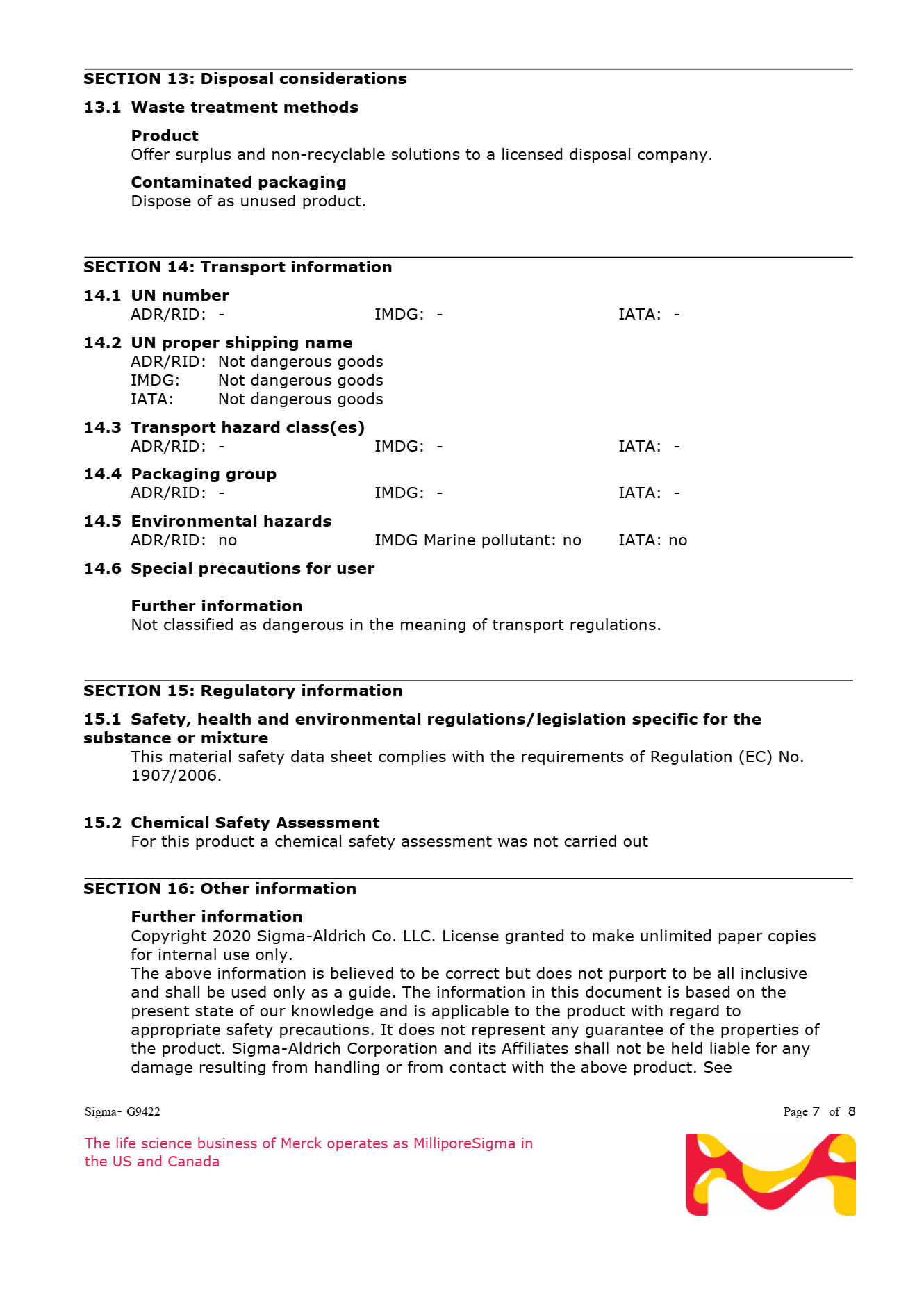


***Supplementary File 4 – Certificate of Analysis: β-Glycerophosphate disodium salt hydrate (Batch SLCP2510)***


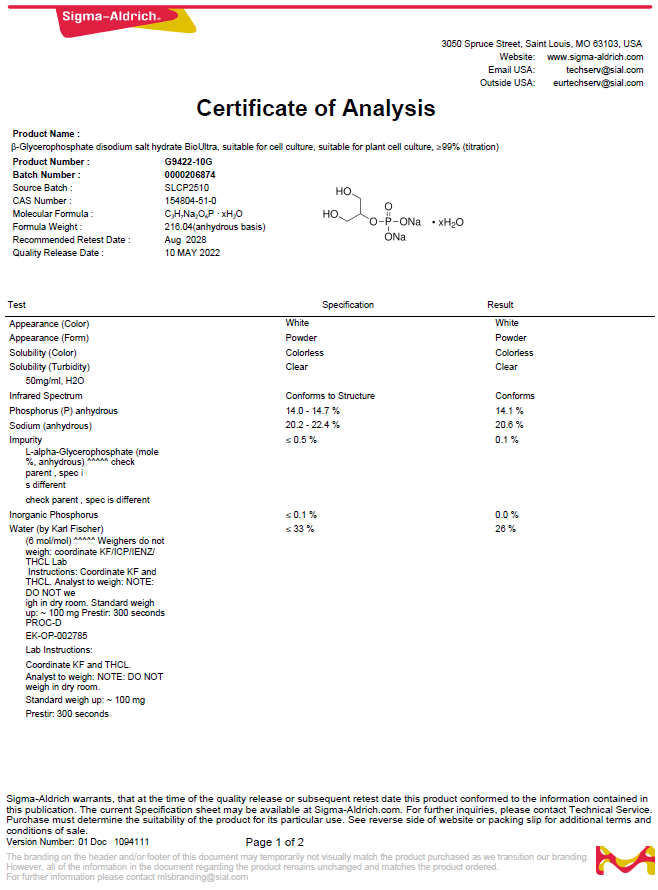


**
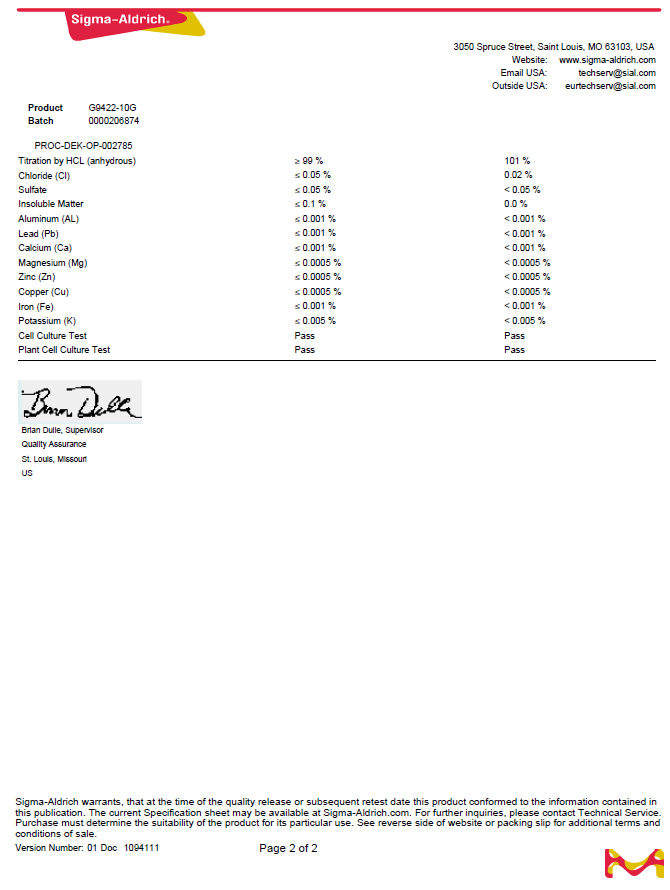
**

***Supplementary File 5 – USP Monograph: Ascorbic Acid***

**
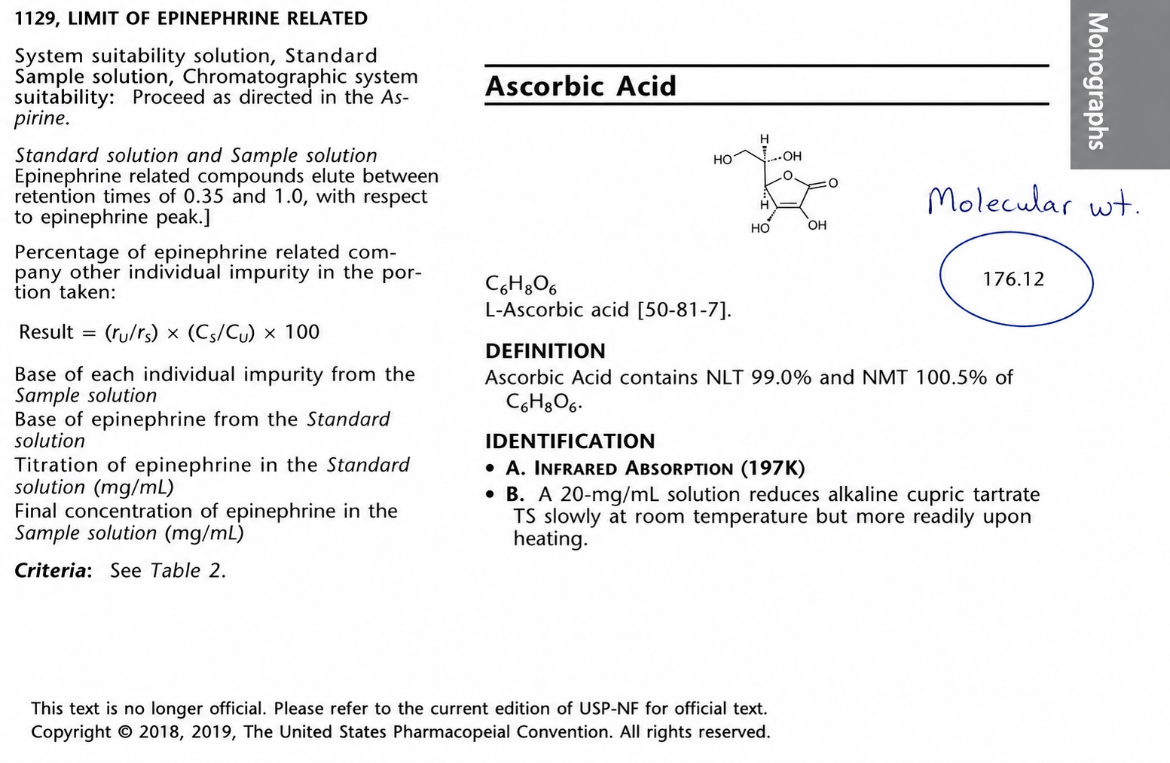
**

**
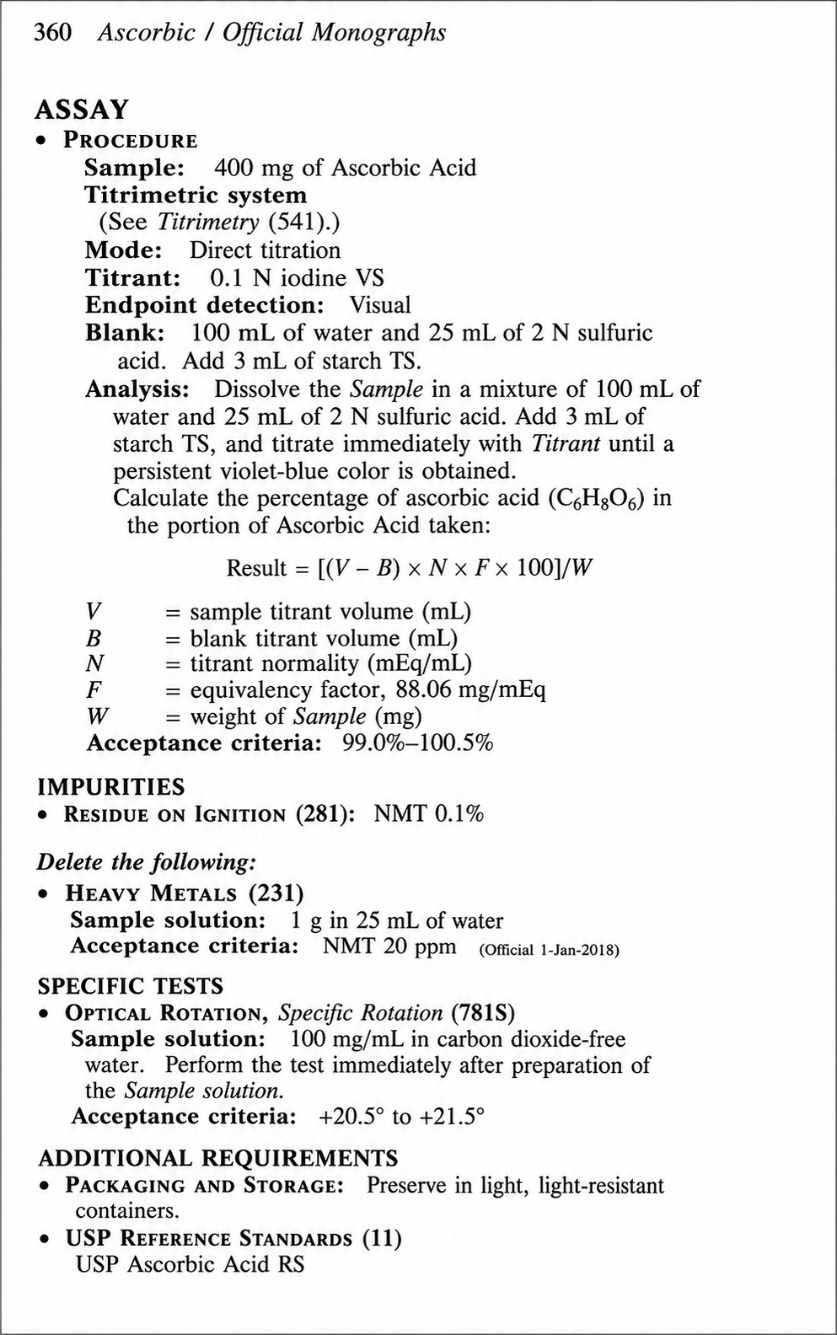
**
